# Photoemission and photoionization time delays and rates

**DOI:** 10.1063/1.4997175

**Published:** 2017-12-15

**Authors:** L. Gallmann, I. Jordan, H. J. Wörner, L. Castiglioni, M. Hengsberger, J. Osterwalder, C. A. Arrell, M. Chergui, E. Liberatore, U. Rothlisberger, U. Keller

**Affiliations:** 1 Department of Physics, Institute of Quantum Electronics, ETH Zurich, 8093 Zurich, Switzerland; 2 Laboratorium für Physikalische Chemie, ETH Zürich, Vladimir-Prelog-Weg 2, 8093 Zurich, Switzerland; 3 Department of Physics, University of Zurich, 8057 Zürich, Switzerland; 4 Laboratoire de Spectroscopie Ultrarapide (LSU), and Lausanne Centre for Ultrafast Science (LACUS), ISIC-FSB, Ecole Polytechnique Fédérale de Lausanne (EPFL), 1015 Lausanne, Switzerland; 5 Laboratory of Computational Chemistry and Biochemistry, Ecole Polytechnique Fédérale de Lausanne (EPFL), 1015 Lausanne, Switzerland

## Abstract

Ionization and, in particular, ionization through the interaction with light play an important role in fundamental processes in physics, chemistry, and biology. In recent years, we have seen tremendous advances in our ability to measure the dynamics of photo-induced ionization in various systems in the gas, liquid, or solid phase. In this review, we will define the parameters used for quantifying these dynamics. We give a brief overview of some of the most important ionization processes and how to resolve the associated time delays and rates. With regard to time delays, we ask the question: how long does it take to remove an electron from an atom, molecule, or solid? With regard to rates, we ask the question: how many electrons are emitted in a given unit of time? We present state-of-the-art results on ionization and photoemission time delays and rates. Our review starts with the simplest physical systems: the attosecond dynamics of single-photon and tunnel ionization of atoms in the gas phase. We then extend the discussion to molecular gases and ionization of liquid targets. Finally, we present the measurements of ionization delays in femto- and attosecond photoemission from the solid–vacuum interface.

## I. INTRODUCTION

Ionization removes one or more electrons from a physical system and is an important fundamental process in nature and technology. Electrons can be removed from their parent system through diverse mechanisms. In this review article, we concentrate on ionization induced by light which is important in biology, photo-chemistry, and science in general. It lies at the basis of techniques for determining the energetic structure of solids and molecules, which by themselves yield important information for technological applications.^1–3^ While we briefly review different light-driven ionization mechanisms, the main focus of this paper will be on ionization dynamics.

## II. IONIZATION PROCESSES DRIVEN BY LIGHT

**FIG. 1.**
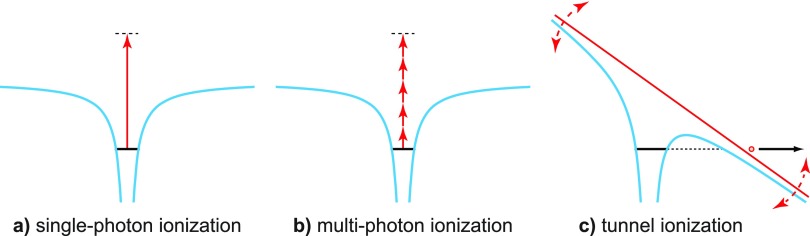
Basic ionization processes in atoms. (a) In single-photon ionization, the atom is ionized through the absorption of a single energetic photon. (b) If the laser intensity is high enough, multiple photons can be absorbed simultaneously and lead to ionization even if the energy of the individual photons is not sufficient. (c) In tunnel ionization, the laser light in the dipole approximation can be considered a classical field that is strong enough to bend the Coulomb potential of the atom, such that a tunnel barrier is created and the electron may tunnel out.

The most basic ionization process that exists even in the simplest bound electronic system, a hydrogen atom, is single-photon ionization [Fig. 1(a)]. A single photon can remove an electron from a physical system if the photon energy is high enough to promote the electron from its initial bound state into the vacuum. Single-photon ionization is also the mechanism underlying the photo effect,^4^ which played an essential role in the early days of quantum mechanics.^5^ Neither single-photon ionization nor any of the other ionization effects can be understood without quantum mechanics.

If we move from atomic hydrogen to systems with more than one electron, ionization processes can quickly become much more complicated. Through correlation effects (i.e., “particles interact with each other”), electrons may share the energy from the absorbed photon, especially when its energy is well in excess of the minimum required ionization energy. This can lead to the excitation or removal of a second electron. This, for example, happens in the Auger effect, where a deep lying (strongly bound) electron is removed. Relaxation of an electron into the newly created vacancy can then lead to the ejection of a second, higher lying electron—the so-called Auger electron.

Besides the increased complexity of an electronic system, also the high light intensities available from laser sources can lead to additional ionization pathways. The most straightforward extension of single-photon ionization that can occur at high light intensity is the ionization through simultaneous absorption of multiple photons [Fig. 1(b)]. It is important to note that in multi-photon ionization, the energy of a single photon can amount to only a fraction of the minimum energy needed for removal of an electron, but with sufficient intensity, it will still occur. Simultaneous absorption of tens or even hundreds of photons is not uncommon.

If we consider the intense laser light an electromagnetic wave rather than a stream of photons, one sees that in the dipole approximation, the electric field in a strong laser beam can bend the Coulomb potential such that an electron can tunnel through the created barrier out into the vacuum [Fig. 1(c)]. This mechanism is called tunnel ionization. At even higher laser intensities, the barrier can be lowered below the ground state of the system, which leads to the so-called above-barrier or over-the-barrier ionization. This model is ultimately limited at higher intensities, but also in the limit of long and toward short wavelengths by the breakdown of the electric dipole approximation.^6,7^

Whether the wave nature or the photon nature of light dominates the ionization mechanism can be determined through the Keldysh parameter $\gamma =\sqrt{I_{p}/2U_{p}}.$^8^ This parameter compares the ionization potential $I_{p}$ with the quiver or ponderomotive energy $U_{p}$ of the electron in the oscillating electro-magnetic light field. For γ≪1, one usually expects the wave nature (or tunneling) to dominate, while for γ≫1, the photon (multi-photon) picture prevails.

It is easy to see that with an oscillating light field or with the simultaneous availability of multiple photons, ionization mechanisms can become much more complicated—in particular, in multi-electron systems. For example, the electron emitted through tunnel ionization may be further accelerated in the oscillating laser field and driven back to its parent ion. Its recollision with the ion may lead to the removal of an additional electron. In multi-photon ionization, on the other hand, the mechanism might take place via an intermediate resonant state. Such a resonance can dramatically enhance the ionization yield for a given photon energy and light intensity.

The overview in this section gives only a rather coarse and incomplete picture of the rich zoo of ionization mechanisms. However, it sets the stage for a much more detailed discussion of the state-of-the-art research in ionization and photoemission dynamics outlined below. The ionization rates of atoms or simple molecules in the gas phase have been understood long ago. However, the time delay of the ionization process itself or the dynamics in more complex systems, liquids and solids, possibly involving cascades of complex interactions, are a hot topic of current research. Recent progress in attosecond pulse generation (1 attosecond = 10^−18 ^s) and extreme ultraviolet (XUV) experimental techniques has allowed us to study such fundamental dynamics in quantum mechanics. In the following, we want to convey some of our excitements in modern time-resolved photoionization and photoemission research to the readers.

## III. DYNAMICS OF IONIZATION

Before we delve more deeply into the topic of ionization dynamics, we want to define the relevant terminology and underlying concepts. How long is an ionization event? While this question sounds simple, one has to clearly define what one means by “how long” or “how fast” to prevent a misunderstanding. Ionization is inherently a quantum mechanical process and quantum mechanics gives us statistical or probabilistic descriptions. Therefore, an ionization rate can be easily determined and has a clear meaning. With regard to the specific time delay of a single ionization process, there remains a heated debate. With some experts arguing that because time is not an observable in quantum mechanics, such questions are not allowed to be asked. Other experts, on the other hand, argue that we should simply follow the electron wavepackets and their group delays will determine the ionization delay. As we will show later, this is not always true and can lead to misleading results because there is no “conservation law” for the peak or the center of the wavepacket.

Ionization dynamics is quantified with lifetimes, rates, and delays. A lifetime is a concept that only makes sense for an ensemble of systems and that is defined in the simplest case with an exponential decay law. If we start with $N_{i}$ systems in a given initial state at a time t=0, we will find $N(t)=N_{i}\cdot e^{-t/\tau_{L}}$ being still in that state after a time t>0. The lifetime $\tau_{L}$ is then defined by the time it takes until the population decayed to 1/*e* of its initial size. A lifetime is, for example, a good concept to describe the ionization dynamics of the Auger electron. The electron that fills the vacancy after removal of a first electron through an energetic photon relaxes spontaneously into that vacancy, following an exponential decay law. The Auger electron is ejected hand in hand with this relaxation and thus ‘inherits’ the exponential law from the relaxing electron.

For such transitions that follow an exponential decay law, we can also quantify the dynamics in terms of ionization rates, i.e., the number of ionization events per unit time. For the simple exponential decay law given above, the associated rate would be $\gamma =1/\tau_{L}$ and the exponential law can be rewritten as $N(t)=N_{i}\cdot e^{-\gamma t}$. In this context, lifetimes and rates are therefore strictly linked and describe the same dynamics.

Finally, one may also ask how long it takes to remove an electron from a system in single- or multi-photon ionization. A concept that partially succeeds in describing these dynamics is the Wigner (or Eisenbud-Wigner-Smith) photoionization delay.^9,10^ The Wigner delay is calculated from the phase shift of the wavefunction describing a particle emerging from a given potential with respect to the wavefunction of a freely propagating particle with the same final kinetic energy. A time is obtained by taking the energy derivative of this phase shift. This is in analogy to the concept of a group delay that is familiar from other contexts of wavepacket propagation (e.g., the propagation of ultrashort optical pulses). The time delay is thus linked to the associated group velocity of the wavepacket. As we will show later in this review, this is, however, not always a successful concept.

A rate can also be defined for tunnel ionization—the tunneling rate. Again, the tunneling rate describes the number of tunnel ionization events per unit time for an ensemble of systems. Given that tunnel ionization can be driven by the electric field of the laser as shown in Fig. 1(c), this rate depends directly on the instantaneous field strength and thus oscillates quickly with time. However, care has to be taken, as there are formulations of the tunneling rate in the literature that are averaged over a laser oscillation cycle.

In addition, a tunneling time can be defined. However, this is much less straightforward than the definition of a lifetime in an exponential decay and different proposals for the definition of such a time exist.^11,12^ There is no direct link of such a tunneling time to the concept of the tunneling rate. Using the attoclock technique^11,13^ we have had excellent agreement with two tunneling theories. However, these results obtained for helium are still debated because they have been based on the single-active electron approximation and thus do not consider electron correlation effects. In addition, even the state-of-the-art time-dependent Schrödinger equation (TDSE) calculations do not resolve this issue.^14,15^ Currently, experiments on atomic hydrogen targets are ongoing which hopefully will resolve this fundamental question in quantum mechanics.

## IV. IONIZATION DYNAMICS IN ATOMS

As the fundamental building blocks of matter, atoms represent the simplest systems that can be ionized. In the following, we therefore cover the ionization dynamics of atoms before we move to molecules and solids in the later sections. As we discuss the ionization dynamics of atoms, we will also introduce the main techniques for their measurement. These methods are later extended toward the more complex systems.

### A. Single-photon ionization of atoms

If the photon energy exceeds the ionization potential of an atom, single-photon ionization can occur. The most established methods to study its dynamics are attosecond streaking^16,17^ and reconstruction of attosecond beating by the interference of two-photon transitions (RABBITT^18,19^), which are both two-color pump-probe schemes employing XUV attosecond pulses and a femtosecond infrared (IR) probe pulse. In the case of streaking, the XUV light is composed of a single attosecond pulse,^20,21^ while RABBITT uses a short train of attosecond pulses evenly spaced in time and with a femtosecond envelope.^19^

Attosecond streaking and RABBITT have been used to measure the relative photoemission time delay between electrons originating from two distinct energy levels of argon^22^ and neon.^23^ Using a coincidence detection technique, we extended these measurements to gas mixtures, which allowed us to time the relative photoemission delay between two different atomic species.^24–26^ Furthermore, we experimentally tested whether the two measurement techniques yield the same delays.^26^

In both measurement techniques, the XUV and IR light are focused into a gas target and the created photoelectron spectra are recorded as a function of pump-probe delay. In the case of RABBITT, the time delay in the atomic photo ionization process is encoded in the phase of oscillating sidebands (SB) that are generated by the interference of two quantum paths that both involve the absorption of a harmonic from the XUV frequency comb of the attosecond pulse train (APT) and the absorption or emission of an additional IR photon.^27^ The total phase of each SB has, however, several contributions. The two main contributions are the difference of the phases of the two harmonics nearest to the SB and the so-called atomic phase. The former originates from a possible (average) chirp of the attosecond pulses within the APT, while the latter contains the phase information from the ionization process. These two main contributions can in general not be separated from each other in a single measurement. Thus, a simultaneous reference measurement from a different state of the same atom^22^ or a different species has to be performed.^26,28^ Subtraction of the phases from the two different states or species yields the difference of the respective atomic phases, while the identical APT-specific phase cancels out. It is important to note that this argument only holds if sidebands created by the same harmonics (same absorbed XUV photon energies) are compared. The information about the photoionization dynamics and in particular, the phase originating from the Wigner delay, is contained in the atomic phase. At most photon energies and in most systems, the Wigner delay is the dominating contribution to this term. An additional phase term is measurement-induced and referred to as the continuum-continuum phase.^22,29^ It originates from the additional infrared-induced transition in the presence of the Coulomb potential of the ion that is required to promote the photoelectron to a final energy within an oscillating RABBITT sideband (i.e., absorption or emission of one infrared photon). The continuum-continuum phase is a universal quantity that is independent of the details of the electronic structure of the probed system. It only depends on the final momentum of the photoelectron, the infrared laser frequency, and the charge of the ion.^29^

In attosecond streaking, the final momentum of the electron photoionized by the attosecond pulse is modulated through interaction with a few-cycle IR probe pulse. The amount of momentum shift depends on the pump-probe delay and follows the (negative) vector potential of the IR pulse at the release time of the electrons. The resulting final energy modulation corresponds to a mapping of time to energy.^17,30,31^ The full phase information of the XUV attosecond pulse can be recovered through a retrieval algorithm called FROG-CRAB (frequency-resolved optical gating for complete reconstruction of attosecond bursts^32,33^). As in the case of RABBITT, this phase contains contributions from the XUV pulse, the pumped transition and the measurement process. The measurement induced counter-part to the continuum-continuum phase appearing in RABBITT is the Coulomb-laser-coupling phase contribution in streaking.^34^ In the attosecond streaking picture, it can be considered a correction to the delay that is due to the deformation of the long-range Coulomb potential by the electric field of the infrared probe laser. The Coulomb-laser-coupling contribution is universal and depends on the same parameters as the continuum-continuum phase.^34^

In analogy to RABBITT, relative timing information was obtained in streaking experiments by comparing the traces from different states of the same target or different target systems. In Ref. 23 a time delay in atomic photoemission of about 20 as has been extracted between electrons ionized from the 2s shell of Neon with respect to ionization from the 2p shell, whereas in Refs. 24–26, the photoemission delays from ground state Argon and Neon were compared.

#### 1. Experimental comparison of RABBITT and streaking

As explained above, both, RABBITT and streaking, are two-color pump-probe schemes involving XUV attosecond and IR femtosecond pulses. In both cases, the XUV radiation is comparably weak and can therefore be described in terms of linear optical interactions. In linear optics, the superposition principle fully holds. In this sense, an attosecond pulse train, as used in RABBITT, is nothing else than a linear superposition of several isolated attosecond pulses, as used in streaking, at equidistant time intervals from each other. As such, one would expect that a RABBITT signal can be constructed by a coherent superposition of streaking traces originating from the individual pulses in the pulse train. Indeed, a RABBITT trace can be constructed in this way.^35^

Given that in a practical experiment, the typical IR intensities used for RABBITT are roughly one order of magnitude smaller than those used in streaking, it is still not obvious that the two methods yield equivalent results, as has been predicted in a theoretical study.^36^

We compared and verified the accuracy of single-photon photoemission delay measurements using both, the RABBITT and the streaking technique.^26^ For this, we measured the relative photoemission delay between electrons originating from the Ne 2p and the Ar 3p ground states. In order to resolve the energy dispersion of this relative delay, we require a relative group delay accuracy on the order of a few tens of attoseconds. This is challenging and requires a good signal-to-noise ratio as well as good knowledge of potential systematic error sources.

Our experiments were performed with a set-up consisting of a front-end capable of producing single attosecond pulses and pulse trains and a cold target recoil ion momentum spectroscopy (COLTRIMS^37^) system.^24^ The APT or single pulse is focused into a gas jet that provides a mixture of argon and neon for simultaneous measurement on both species under identical experimental conditions. The detected photoelectrons and the corresponding RABBITT and streaking traces are assigned to the two species in data post-processing, using the coincidence information from COLTRIMS.

**FIG. 2.**
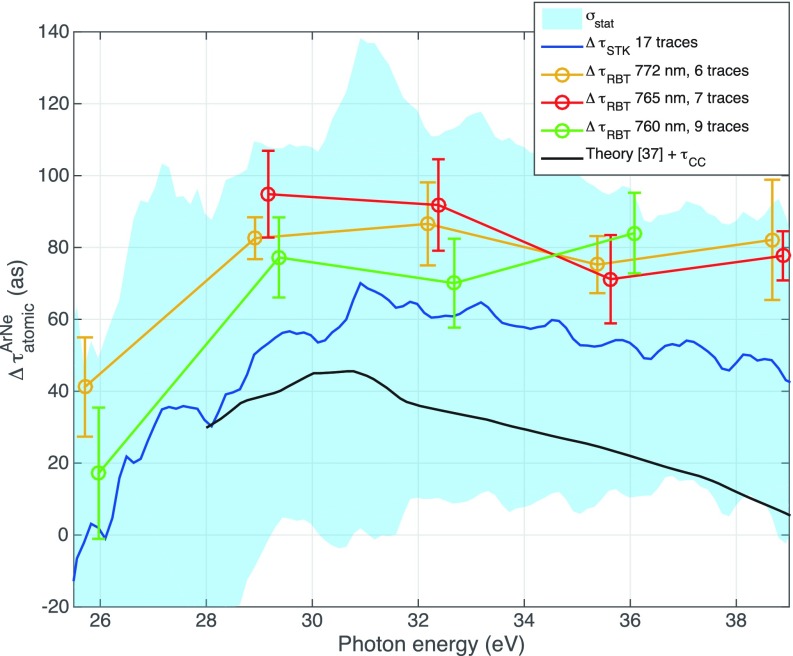
Comparison of RABBITT and streaking data showing the relative photoemission delay between ground state Ar and Ne. The three different sets of RABBITT data have been taken with slightly different infrared wavelengths. Within the experimental uncertainty, all measurements agree. For comparison, a theoretically calculated Wigner delay in the continuum is shown according to Ref. 38. Adapted with permission from Cattaneo *et al.*, Opt. Express **24**, 29060 (2016).^26^ Copyright 2016 Optical Society of America.

The experimental data from the streaking measurements and three RABBITT scans performed with slightly different laser wavelengths are shown in Fig. 2. They are also compared with a theoretical prediction taken from Ref. 38. Within the uncertainty of the experiments, the two methods yield comparable results. However, there appears to be a small disagreement between the experiment and the simple Wigner delay. The discrepancy was explained by the presence of a number of atomic resonances in Ar at excitation photon energies between 25 and 30 eV^39^ which affect the photoemission delay.^25^ The reason why these sharp resonances have a “washed out effect” in the experiment might be two-fold. In the case of RABBITT, our APT XUV spectrum is not resonant with any of the resonances in this range. In the case of streaking, on the other hand, the IR intensity is about an order of magnitude higher than for RABBITT and significantly modifies the lifetimes of the resonances, which might wash out the associated features in the measured photoemission delay. It was shown, however, that RABBITT can resolve such a resonance by scanning the probing XUV photon energies across the resonance.^40^

More details on the comparison of RABBITT and streaking measurements, including the role of the chirp of the XUV pulses as an important systematic error source in phase extraction from streaking, are presented in Ref. 26.

#### 2. Angular anisotropy in photoemission from helium

When interpreting the photoemission delays from RABBITT and streaking data, one has to be aware what these methods actually measure. Both techniques are two-color pump-probe schemes. In the case of RABBITT in particular, which can conveniently be understood in the photon picture, it is clear that the measurement process is a two-photon mechanism: absorption of an XUV photon and absorption/emission of an infrared photon.

**FIG. 3.**
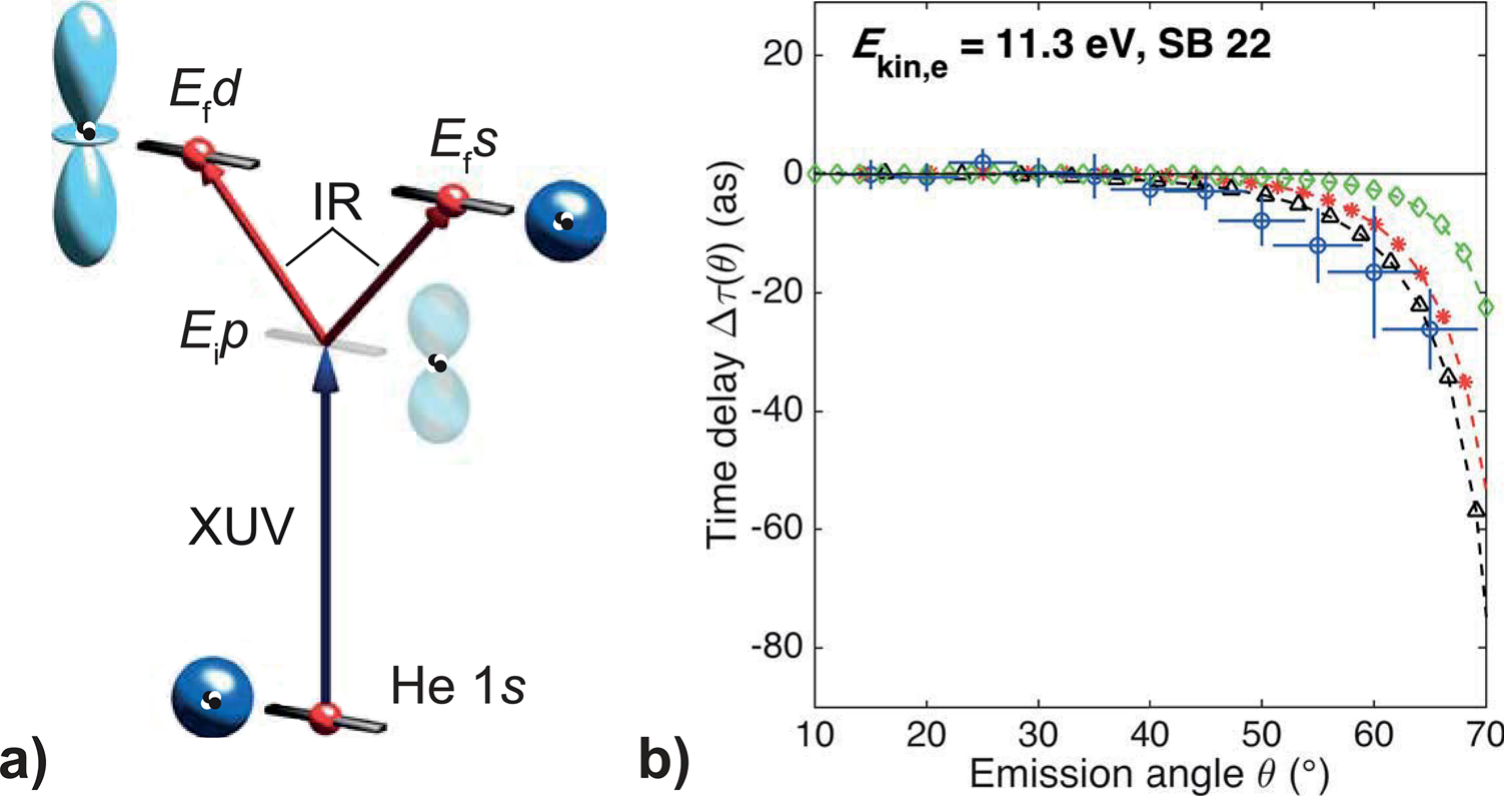
Photoemission delay anisotropy resulting from the two-photon measurement process. (a) In the RABBITT measurements on helium, the XUV photon excites the electrons from the ground state with s-symmetry to a continuum wavefunction with p-symmetry. The infrared probe field promotes the electron further into either a s- or d-wavefunction. The observed anisotropy can be understood from the angle-dependent mixing of these two channels. The angular dependence is a result of their differing spatial symmetry. (b) Measured (blue circles) and calculated photoionization time delay as a function of angle with respect to the polarization axis of the XUV and infrared pulses. The calculated black and red curves are based on solving the time-dependent Schrödinger equation (TDSE), nearly exact and with single-active electron approximation, respectively,^41^ the green curve is based on the lowest order perturbation theory.^42^ Reprinted with permission from Heuser *et al.*, Phys. Rev. A **94**, 063409 (2016).^43^ Copyright 2016 American Physical Society.

In recent experiments, we measured the angular dependence of the single-photon photoemission delays from ground-state helium with respect to the polarization axes of the exciting XUV light and the probing infrared beam using the same attosecond COLTRIMS apparatus described above.^24,43^ Given the s-symmetry of the ground state helium atom, one might expect that the photoemission delay is fully isotropic and does not exhibit an angular dependence. However, we could show that the two-photon nature of the RABBITT process yields a superposition of s- and d-like continuum wavefunctions, which results in a strong angular dependence of the measured delay^43^ (see Fig. 3). The absorption of the XUV photon promotes the electron from its initial s-symmetry to a continuum wavefunction with p-symmetry. Absorption and emission of the probing infrared photons then yield a wavefunction of either s- or d-symmetry. Electrons from these two dominant pathways are superimposed for a given final electron energy. Due to the different spatial symmetry, the mixing ratio of these two channels depends on the detection angle with respect to the polarization axis, which leads to the observed angular dependence of the photoemission time delay.^43^ Particular care has therefore to be taken when interpreting angularly integrated photoemission delay data. The integration might conceal an angular dependence and lead to a systematic error in the retrieved delay.

#### 3. Dependence of photoionization delay on electronic fine-structure

**FIG. 4.**
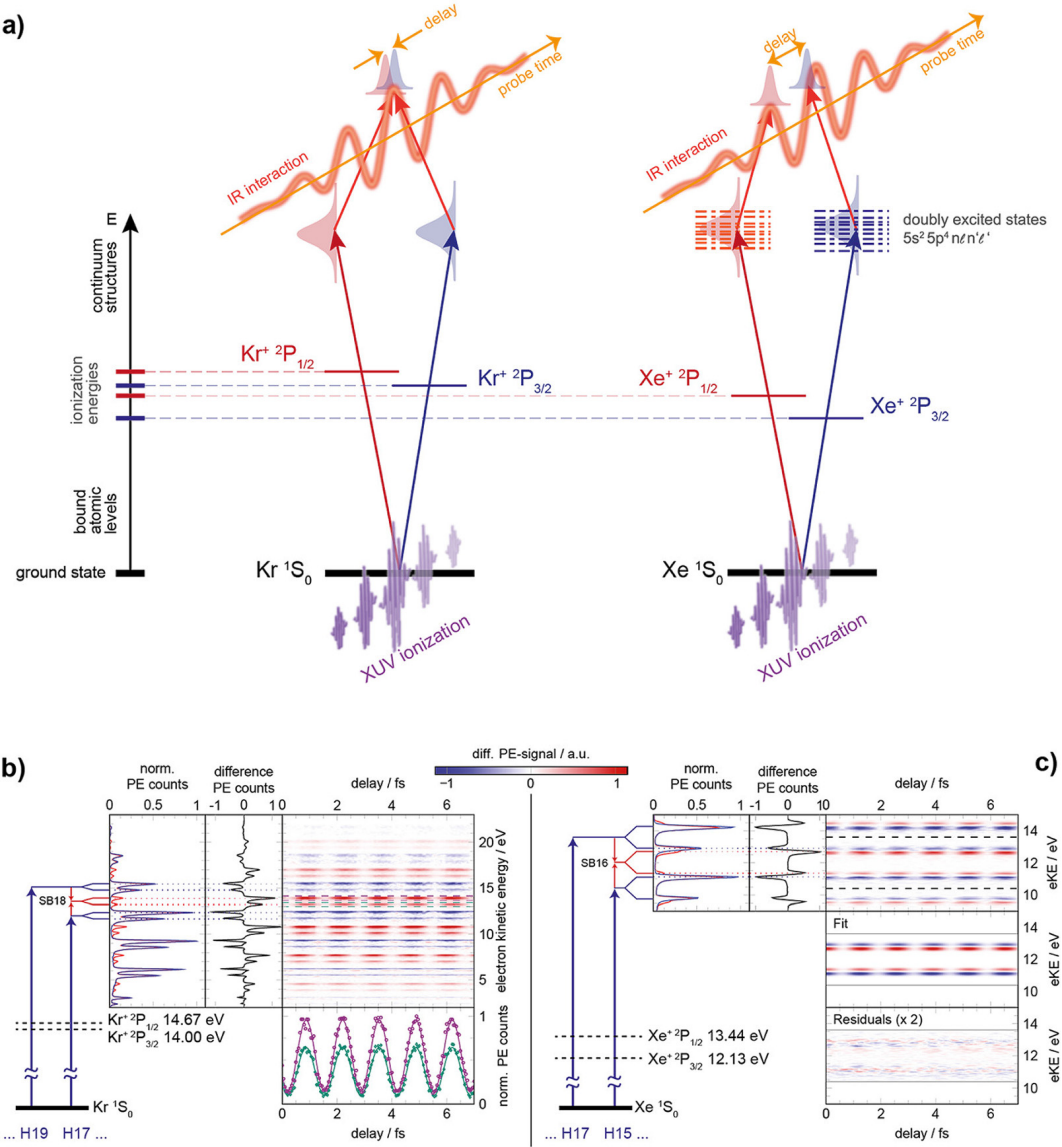
Configuration for measurement of fine structure effects on photoemission delays (a). The relative photoionization delay of photoelectron wavepackets associated with the ^2^P_3/2_ and ^2^P_1/2_ final states of Kr^+^ (b) and Xe^+^ (c) are measured. The neutral atoms are ionized by an XUV attosecond pulse train, superimposed with an infrared pulse at a wavelength of 800 nm. The differential photoelectron spectra in the presence and absence of the infrared pulse are recorded on a single-shot basis. In the case of Xe, the delays are extracted using a two-dimensional fitting procedure. Reprinted with permission from Jordan *et al.*, Phys. Rev. A **95**, 013404 (2017).^44^ Copyright 2017 American Physical Society.

So far, we have discussed photoionization delays between electrons leaving an atomic cation in different electronic states. One may therefore ask: is there any delay between photoelectrons leaving the ion in different fine-structure levels of the same electronic state? This question has been addressed experimentally in Ref. 44. Photoionization delays have been measured between electrons leaving a rare gas cation (Kr^+^ or Xe^+^) in the ^2^P_3/2_ and ^2^P_1/2_ levels of their respective electronic ground states. These measurements have been carried out using attosecond interferometry with a high-resolution photoelectron spectrometer capable of resolving the fine-structure splittings in both cations throughout the XUV spectral range and using advanced single-shot data acquisition techniques (Fig. 4).

These measurements, carried out between photon energies of 18 and 40 eV, have revealed small delays in the case of Kr, lying systematically below 8 as in magnitude. This is in remarkable contrast to a previous measurement,^45^ which found much larger delays. In the case of Xe, which was not investigated previously, surprisingly large delays (τ_3/2_-τ_1/2_) have been measured, reaching from −9±4 as at 21.7 eV to +33±6 as at 33.4 eV.

Importantly, these measurements show that delays caused by fine-structure effects are not, in general, negligible compared with delays associated with different electronic states. This insight is expected to extend from atoms to molecules and solids. Spin-orbit delays are expected to be particularly important in systems containing heavy elements because spin-orbit coupling is a relativistic effect.

These measurements have also been used to verify the accuracy of state-of-the-art theories for attosecond photoionization delays.^44^ The results have been compared to time-dependent configuration-interaction singles (TD-CIS) calculations,^46^ an explicitly time-dependent method that has been used to simulate the experiment without additional approximations. The measured delays have also been compared to the random-phase approximation method, a time-independent method that is renowned for achieving near-quantitative accuracy in the calculation of photoionization cross sections and asymmetry parameters (see, e.g., Ref. 38 for a recent example). This method has been combined with an analytical treatment of the continuum-continuum delays, which enabled a detailed study of this approximation.^44^

Whereas both theories are in good agreement with the very small delays measured in the case of Kr, significant discrepancies were found in the case of Xe. A detailed consideration of the possible origins of this discrepancy led the authors to conclude that an incomplete description of electron correlation, in particular of the interaction between singly- and doubly-excited configurations in both methods, was the most probable origin of the deviation between the experiment and theory.

#### 4. Limits of intuitive Wigner delay picture

Above, we have introduced the Wigner delay that is defined as the energy derivative of the phase of an electron escaping the potential of its parent ion with respect to the phase of a free electron with the same final kinetic energy. As already mentioned, the propagation of the electron wavepacket is analogous to the propagation of a laser pulse through a dispersive medium: The derivative of the spectral phase *ϕ* at any frequency *ω* (or energy) yields the group delay τ=∂ϕ/∂ω that in the absence of absorption describes how much time it takes for the peak of the pulse to traverse a certain distance in a medium. In the case of the quantum mechanical wavepacket, the Ehrenfest theorem links this description to the classical picture of a particle moving at its group velocity.

While the Wigner delay is always a well-defined quantity from a mathematical perspective, care has to be taken when interpreting it in terms of this simple semiclassical picture. As is the case for optical pulses, the interpretation of a group delay is less straightforward if the wavepacket undergoes a significant reshaping through an energy filter. If the propagating wavepacket experiences resonances or strongly energy-dependent damping, there is no intuitive link between the maximum of the wavepacket before this filter and its maximum after the filter—the two points in the wavepacket have no physical relationship. As such, the group velocity (“the speed of the center of the wavepacket”) can assume almost any numerical value—for example, become superluminal.^47^ This is the reason why the Wigner delay is not a good concept to describe ionization delays in the tunneling regime^11^ and in single photon emission with autoionizing states.^25^ Electron wavepackets in contrast to photons even disperse in vacuum and become strongly chirped during propagation. For example, a tunnel barrier is a very strong energy filter that prevents a simple relationship between a wavepacket maximum on one side of the classically forbidden region and of the maximum of the wavepacket after tunneling through the barrier [see also Fig. 6(b)].

Similarly, in single photon ionization when an electron is liberated into a non-resonant continuum, the measured delay is well described by the Wigner delay which gives a direct link to the classical propagation delay with the center of the electron wavepacket. However, the situation becomes more complicated when the autoionization states are involved in the single-photon ionization. For example, with argon, we observed that the Wigner phase delay is affected by them.^25^ Furthermore, most recently, we could show that these autoionization resonances in argon not only distort the phase of the emitted photoelectron wavepacket but also introduce an angular dependence.^48^ These strong angular-dependent phase distortions make it very difficult to directly link the Wigner delay to an equivalent classical trajectory of the photoelectron.

### B. Tunneling delay

In Sec. IV A, we discussed the dynamics of single-photon ionization. But how long does it take to remove an electron from an atom in the tunnel ionization regime? In tunnel ionization, an intense low-frequency laser field in the dipole approximation bends the atomic potential sufficiently that a transient tunnel barrier is formed and the electron can escape the atom through tunneling [Fig. 1(c)]. The rate of tunnel ionization is described by the following law:^49^

$$W_{TI}\propto \;\text{exp}\;\left(-\frac{2{\left(2I_{p}\right)}^{3/2}}{3E}\right),$$

where $I_{p}$ is the ionization potential and E the non-adiabatic electric field amplitude.

The dynamics of this process can be resolved with a technique called attosecond angular streaking or the attoclock.^50^ The attoclock uses close-to-circularly polarized laser pulses. It exploits the fact that due to the small remaining ellipticity of the polarization and the exponential dependence of the tunneling rate on field strength, ionization is most probable when the field vector points to the direction of the major axis of the polarization ellipse. In fact, a change of the field amplitude by 10% (from 0.1 a.u. to 0.09 a.u.) will result in a reduction in the ionization rate by almost one order of magnitude.

Measuring time in the attoclock is achieved by counting the field oscillation cycles similar to the operation principle of a regular clock: the rotating field vector acts like the minute hand of a watch, mapping time to angle. In the attoclock, the close-to-circularly polarized laser electric field ionizes and further deflects the electrons in the spatial direction perpendicular to field propagation, mapping the instant of ionization to a final angle of the momentum vector in this plane. This attoclock runs over 360° within one optical cycle that takes about 2.7 fs for a laser pulse centered at 800 nm wavelength. Knowledge of the orientation of the laser polarization ellipse from polarimetry measurements yields the time-zero calibration for the clock: together with the tunnel ionization rate, we know in what angular direction to expect the highest electron count. As the determination of the highest electron yield from the experimental data boils down to a peak search, the corresponding angle can be determined with very high precision significantly below one optical period. For example, for a center wavelength of 735 nm, one degree in the polarization plane corresponds to 7 as. Furthermore, there is in principle no fundamental limit to the precision of determining this most probable ionization delay with peak search, as this is a purely statistical procedure—the better the statistics, the better the precision.

But how do we extract a tunneling delay time or tunnel traversal time from this information? A real and measurable tunneling delay time would manifest itself in an angular offset of the entire momentum distribution compared with its expected orientation with zero tunneling delay time. In the latter case, the electrons would appear “at the end of the tunnel” at the instant of maximum ionization rate or maximum laser field. Any real delay would cause the electrons to appear at an offset with respect to that angular direction. From the knowledge of the orientation of the polarization ellipse, we can calculate where we would expect the maximum electron count for a zero-tunneling-delay-time hypothesis. If all effects acting on the emitted electrons are properly taken into account,^51^ any angular offset must be attributable to the tunnel traversal time.

While the first demonstration of angular streaking on helium yielded no measureable tunneling delay times,^13,51^ later refinements allowed to reduce the error bars and yielded data that could not be explained with instantaneous tunneling.^11^ The attoclock principle was also used to time other ionization processes. It was used to measure the relative delay between the two electrons emitted in sequential double-ionization of argon.^52^

**FIG. 5.**
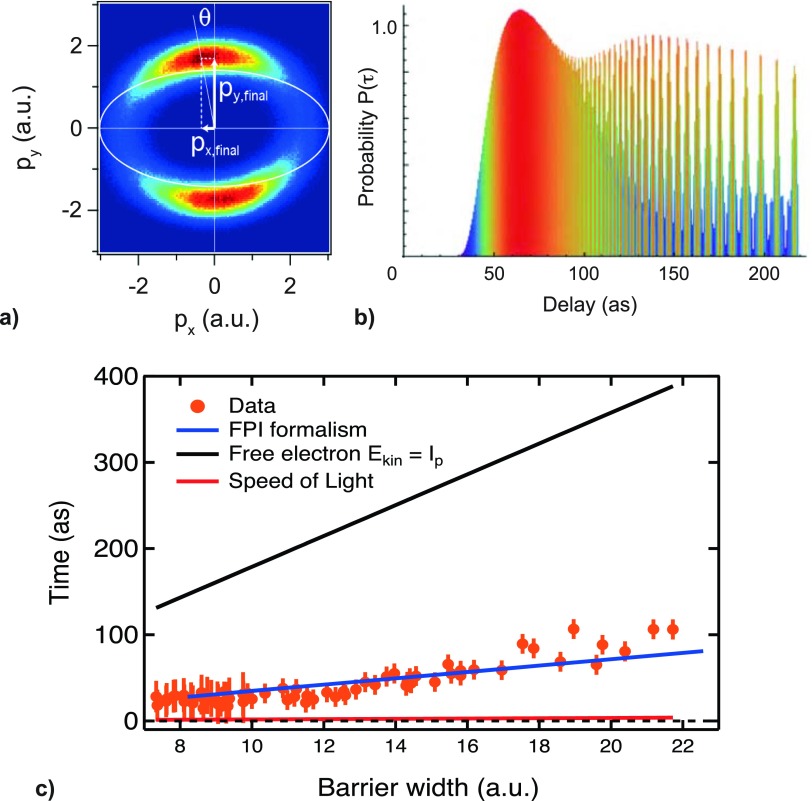
Attoclock technique for measuring the tunnel ionization delay time: (a) Peak search for the highest count of photoelectrons in the polarization plane. (b) Probability distribution for different tunneling times calculated by the Feynman Path Integral formalism.^12^ The peak of this distribution determines the “most probable trajectory” and is consistent with “peak search” in the attoclock measured data (i.e., angle with highest count of electron). (c) Measured tunneling time using the attoclock technique with a helium gas target in the regime of the adiabatic approximation.^11^ Reprinted with permission from Landsman and Keller, Phys. Rep. **547**, 1 (2015).^12^ Copyright 2015 Elsevier.

**FIG. 6.**
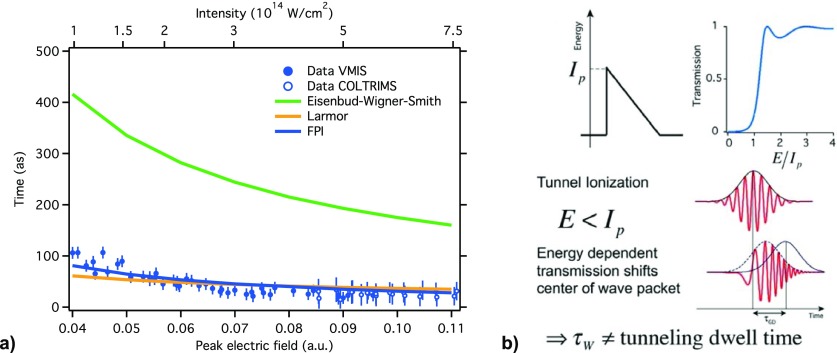
(a) Wigner delay is not in agreement with the attoclock results for tunneling.^11^ (b) Energy filter in tunneling probability makes the chirped electron wavepacket “jump” in time and therefore the group delay ($\tau_{\text{GD}}$) of the peak or the center of the wavepacket cannot represent the time the electrons spend inside the classically forbidden region under the tunnel barrier (i.e., the tunneling dwell time).

With the attoclock technique applied to a He gas target, we had excellent agreement with the Feynman Path Integral (FPI) theory (Figs. 5 and 6) and the Larmor time (Fig. 6). But we clearly did not have a good agreement with the Wigner time. This can be explained by the energy filter of the tunneling probability as shown schematically in Fig. 6(b) and which was also observed in single photon ionization.^25^

### C. Time-dependent density functional theory (TDDFT) based simulations of Attoclock experiments including nonadiabatic and many-electron effects

As explained in Sec. IV B, the attosecond angular streaking technique, also known as “attoclock,”^50^ uses an elliptically polarized laser field of high intensity (of the order of 0.01–1 PW/cm^2^) to induce tunnelling ionization of noble gas atoms. Due to the elliptical polarization of the field, the probability of recollisions and recombinations of the ionized electrons with the parent ions is reduced to a minimum. Temporal information on tunnelling, like for instance, the tunnelling time in single ionization of helium^13^ and argon,^52^ the time delay in the double ionization of argon,^52^ as well as the tunnelling geometry,^51^ and, more recently, information on the adiabaticity of the tunnelling process,^53^ can be inferred from the momentum distributions of emitted electrons and ions.

The interpretation of the experimental momentum distributions is based on a comparison with momentum distributions obtained by semiclassical calculations. In this approach, electron dynamics after tunneling is treated classically as described by the TIPIS model (tunnel ionization in parabolic coordinates with induced dipole and Stark-shift),^51^ which relies on the single active electron approximation (SAE) and includes many-electron effects only implicitly via a static polarizability term.

The time delay in single ionization processes is inferred from the angular offset between the calculated and the measured peak of the distributions^11,12^ (Fig. 5). This interpretation of the angular offset has been the object of debate. A current more detailed invited review article is in preparation which will discuss all the different approximations such as dipole approximation and non-adiabatic effects in the tunnelling process.^53–56^ We could show that the excellent agreement with the Feynman Path Integral formalism as shown in Fig. 5 is still valid without the adiabatic approximation. In the non-adiabatic regime, the laser field strength calibration is, however, affected, which results in an effectively thicker tunneling barrier width in comparison with the adiabatic case shown in Fig. 5(c).^57^ The influence of the single active electron approximation, on the other hand, will have to be resolved with an attoclock measurement on a hydrogen gas target. More sophisticated theoretical models based on a two-electron semi-classical model^58^ and TDSE calculations for hydrogen^14^ and helium^15^ do not resolve the issues.

Explicit time propagation of the electronic orbitals in the framework of time dependent density functional theory (TDDFT) can be a suitable alternative to solutions of the TDSE for model Hamiltonians, as it does not rely on a static approximation of the barrier and correlation as well as nonadiabatic effects are explicitly taken into account.

#### 1. Computational approach

We present here the results of a computational TDDFT-based study of a single hydrogen or argon atom in the presence of a short and intense laser pulse like those adopted in attoclock experiments. In our simulations, the laser field is represented in the velocity gauge by a vector potential described as a monochromatic pulse with Gaussian envelope, G(t)=exp(−t^2^/2σ^2^) rotating in the XY plane of our coordinate reference frame

$$\frac{A}{c}\left(t\right)=\frac{E_{0}}{\omega }\frac{1}{\sqrt{1+\varepsilon^{2}}}G\left(t-t_{0}\right)\left\{-\varepsilon \text{sin}\left[\omega \left(t-t_{0}\right)\right]\hat{x}+\text{cos}\left[\omega \left(t-t_{0}\right)\right]\hat{y}\right\},$$

where ω is the carrier frequency and ε is the ellipticity parameter.

The electron dynamics is described in the propagation-TDDFT framework, in which a set of auxiliary, non-interacting and explicitly time-dependent orbitals, {$\phi_{j}\left(s_{j},t\right)$}, are introduced to reproduce the correct time-dependent density

$$\rho \left(\left\{s_{j}\right\},\;t\right)=\sum_{j=1}^{N_{\text{st}}}{\left|\phi_{j}\left(s_{j},t\right)\right|}^{2},$$

where {**s**_j_} indicates the electronic spatial and spin coordinates {**s**_j_}={**r**_j_,σ_j_}.

The time dependence of these single particle (Kohn-Sham) orbitals is described by the following set of equations:

$$i\partial_{t}\phi_{j}\left(s_{j},t\right)=h_{j}^{\text{KS}}\left[\rho \left(\left\{s_{j}\right\},\;t\right)\right]\phi_{j}\left(s_{j},t\right),\;j=1,\ldots ,N_{\text{st}},$$

in which *N*_st_ are the electronic states and *h_j_*^KS^ are the Kohn-Sham Hamiltonians expressed as functional of the time-dependent density

$$h_{j}^{\text{KS}}\left[\rho (t)\right]=-\frac{1}{2}\nabla_{j}^{2}+V_{H}\left[\rho (t)\right]+V_{XC}\left[\rho (t)\right]+V_{\text{ext}}\left[\rho (t)\right],\;j=1,\ldots ,N_{\text{st}}.$$

The time-dependent Kohn-Sham equations are integrated following the recursive Crank-Nicholson scheme,^59^ as implemented in the CPMD (Car-Parrinello molecular dynamics) code;^60^ the integration time-step is allowed to change during the simulation to adapt to the variations in the field strength and is of the order of δt = 0.12 as. The time-dependent exchange correlation functional was described by the time independent PBE (Perdew, Burke, Ernzerhof) approximation applied to the time-dependent density.^61^ Through the use of the asymptotic correction introduced by van Leeuwen and Baerends (LB94),^62^ the ionization potentials are accurately described. The simulation box is orthorhombic with lengths of 100 Å × 100 Å × 50 Å; the atom is initially placed at the center of the box. Troullier-Martins pseudopotentials^63^ are used to soften the Coulomb repulsion in the proximity of the nucleus and a kinetic energy cut-off of 70 Ry is used for the plane wave basis set expansion.

In the simulation of an ionization process, part of the electronic wave function spreads far from the nucleus: to avoid unphysical reflections of the electronic wave function at the edges of the simulation box, it is necessary to adopt absorbing boundary conditions. We implemented a mask function, which goes smoothly from unity to zero at the edges of the box and multiplies the Kohn-Sham orbitals after each time-propagation step. In the present work, the portion of the box affected by the mask function is at maximum 5 Å from the box sides.

The values of the field parameters used in the present simulation are chosen in such a way as to match typical experimental conditions,^52^ except for the pulse duration that was chosen slightly shorter than the experimental ones. The parameter values are E_0_ = 0.613 GV/cm, ε = 0.78, ω = 2.56 fs^−1^ and σ = 0.72 fs. This corresponds to a pulse of wavelength λ = 740 nm, duration D = 1.7 fs and peak intensity I = 0.5 PW/cm^2^ and to a value of the Keldysh parameter^8^ of γ_Η_ = 0.516 for H and γ_Αr_ = 0.555 for Ar.

**FIG. 7.**
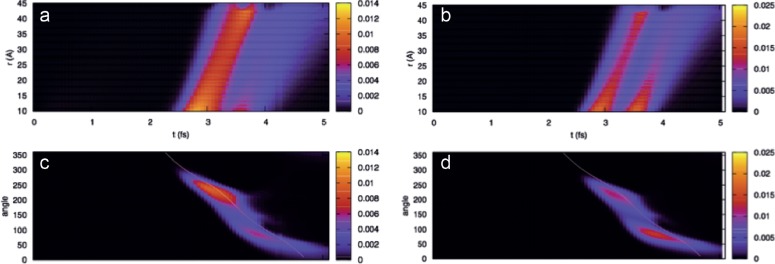
Illustration of the electron dynamics in hydrogen [(a) and (c)] and argon [(b) and (d)]: time behavior of the radial [(a) and (b)] and angular [(c) and (d)] projections of the electronic density. In the angular plots, the direction of the laser field is shown as a yellow line.

As an illustration of the electron dynamics in the H and Ar atoms in the presence of the laser pulse, Fig. 7 shows the time evolution of the radial and angular projections of the electronic density. As expected, before the external field has reached its maximum value, only minor charge polarization is visible. At later times, two ionization bursts become visible, starting in correspondence with the two maxima of the electric field. Part of this charge is soon reabsorbed by the nucleus, while some escapes and is absorbed at the box boundaries. The two bursts are almost equally intense in argon, while in hydrogen, the second one is less pronounced due to saturation effects.

The calculation of ionization probabilities and rates gives a more quantitative description of the ionization phenomenon. Within a TDDFT description of the electronic state, the ionization probability P^+^(t) (in one electron systems) can be obtained by integrating the time-dependent electronic density within a sphere Σ_c_ of radius R_c_ centered on the nuclear position^64^

$$P^{+}\left(t\right)=1-\int_{\Sigma_{c}}dr\rho \left(r,t\right).$$

In many-electron systems an estimate of the orbital ionization probability can be obtained in an analogous manner, assuming single ionization.^64^ The ionization rate w(t) is given by the time derivative of P^+^(t). The sphere Σ_c_ is introduced to separate, albeit in an approximate way, bound states from free-state contributions of the electronic wave function: the electronic density outside the sphere will then be assumed to be “ionized.” When the external field is still present, the results of this procedure depend on the value of R_c_; the asymptotic, zero field values are instead independent of the choice of the surface and can be used to quantify the effect of the laser on the electron dynamics. The cut-off R_c_ is usually chosen so that all the relevant bound states are contained inside the sphere Σ_c_, while the density found outside Σ_c_ is assumed to be due to the states belonging to the continuum. This criterion determines a lower bound to the value of R_c_. In the present calculation, to limit the possible impact of wavefunction polarization on the calculated probabilities, we determined the tunnel exit provided by the TIPIS model for each atomic species (hydrogen and argon) under the same field conditions. This value was then used to define the sphere radius.

**FIG. 8.**
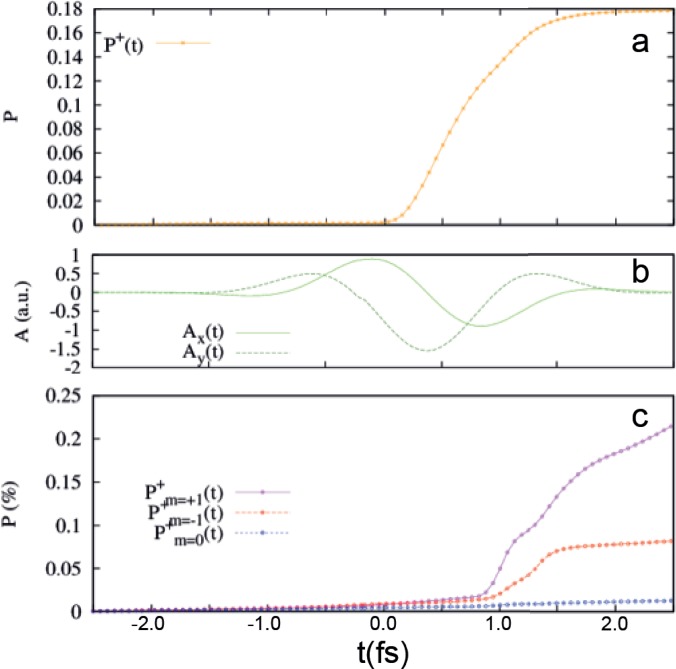
Calculated time-dependent ionization probabilities in (a) hydrogen and (c) argon. For the argon atom, contributions from the different p orbitals are shown separately to highlight the dependence of the ionization probability on the magnetic quantum number m, an effect found theoretically.^67^ X and Y components of the laser vector potential used in the calculations are shown in panel (b).

Panels (a) and (c) of Fig. 8 display the orbital ionization probabilities in H and Ar, respectively, under the effect of the laser field whose vector potential is shown in Fig. 8(b). In both atoms, the probability is zero up until the center of the pulse and saturates at the end of it. In Ar, probabilities are larger for the p orbitals laying in the polarization plane, while electronic charge is removed with smaller probability from the m = 0 p orbital. This result is consistent with other theoretical predictions, based on TDSE solutions of effective one-electron model potentials,^65^ and experimental findings.^66^ The total ionization probabilities at the end of the pulse are of about 18% for H and less than the 1% for Ar.

**FIG. 9.**
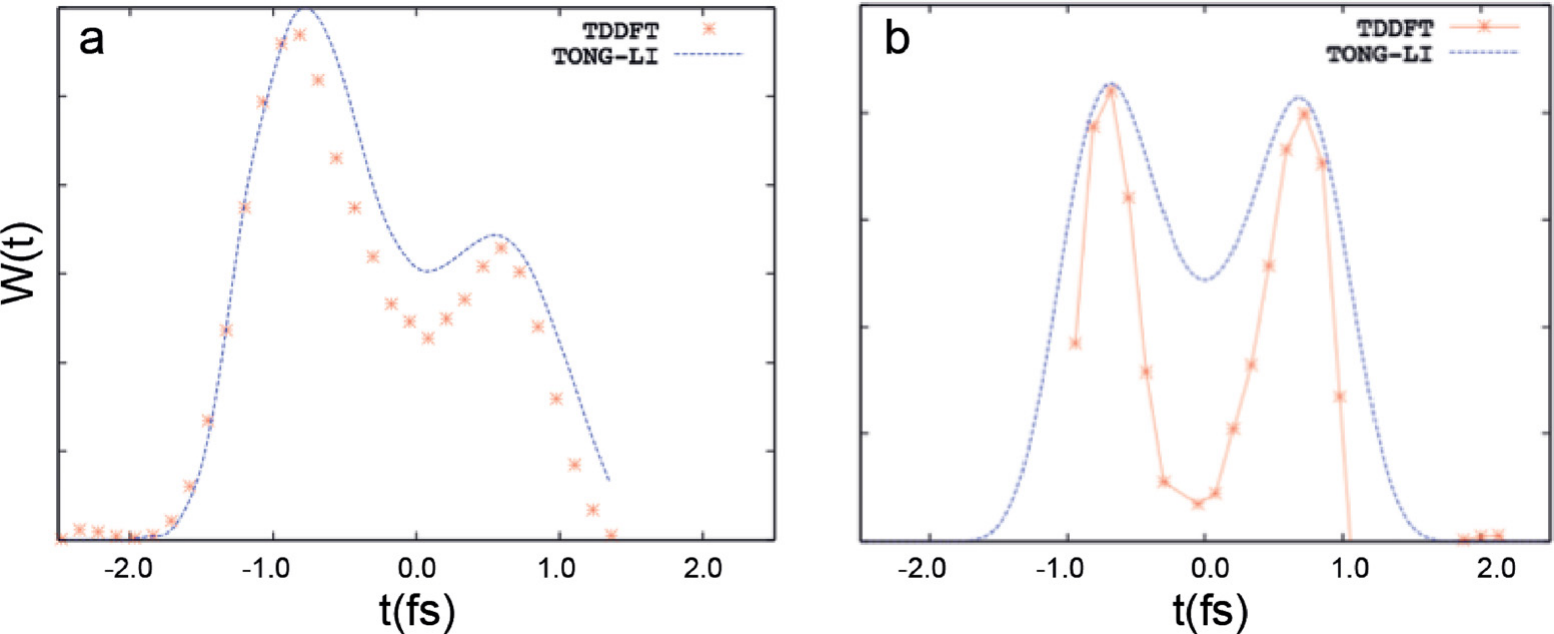
Calculated ionization rates in (a) hydrogen and (b) argon in comparison with the ionization rates predicted by the Tong and Lin model^68^ used in the interpretation of attoclock experiments to determine the initial conditions of semiclassical trajectories.^51^

Figure 9 shows the ionization rates obtained for the H atom [Fig. 9(a)] and the Ar atom [Fig. 9(b)] following the procedure described above, assuming a value of R_c_ corresponding to the tunnel exit calculated according to the TIPIS model. For comparison, we also show the empirical tunnelling rates proposed by Tong and Lin^68^ that are used to provide the initial conditions for the classical electronic trajectories in the TIPIS calculations.^51^ TDDFT and Tong-Lin rates have a qualitatively similar behavior with two peaks corresponding to the ionization burst shown in Fig. 7. For the H atom, due to saturation effects, a marked first ionization peak is followed by a less pronounced shoulder. In Ar, where the ionization probability is much smaller, saturation is far from being reached and the two peaks have nearly identical heights. The main difference between TDDFT and Tong-Lin theory is the deeper depletion between the two maxima in the curves for Ar, while the hydrogen curve is close to the one predicted by Tong-Lin theory. One can speculate that these discrepancies, present only in the many-electron system, can be related to correlation effects.

For a more direct comparison with the attoclock experiments, including a comparison of simulated versus measured momentum distributions, we are currently extending the TDDFT simulations described in this section to pulses of longer duration and including also the He atom as a third system.

## V. IONIZATION DYNAMICS IN MOLECULES

Moving from atoms to molecules significantly increases complexity. This is due to the lower symmetry and additional degrees of freedom of molecules. Early experimental results on H_2_^69^ and CO^70^ are still under further theoretical investigations before a more detailed journal publication can be done. Especially for H_2_, we can no longer neglect the effect of the nuclear motion on the photoionization delay.

The experimental challenges associated with attosecond interferometry of molecules originate from the considerable congestion of molecular photoelectron spectra generated by attosecond pulse trains (APT). This challenge has been overcome by combining spectral filtering of the APT with different thin metallic foils and single-shot data acquisition.^71^ One approach to address the theoretical challenges is based on a complete theory of molecular photoionization delays based on state-of-the-art time-independent molecular scattering calculations.^71,72^

In addition to the attosecond photoemission delays from molecules, we discuss in this section also the measurement of strong-field photoionization rates on molecules, which is a complementary aspect of molecular ionization dynamics.

### A. RABBITT on water vapor and nitrous oxide

**FIG. 10.**
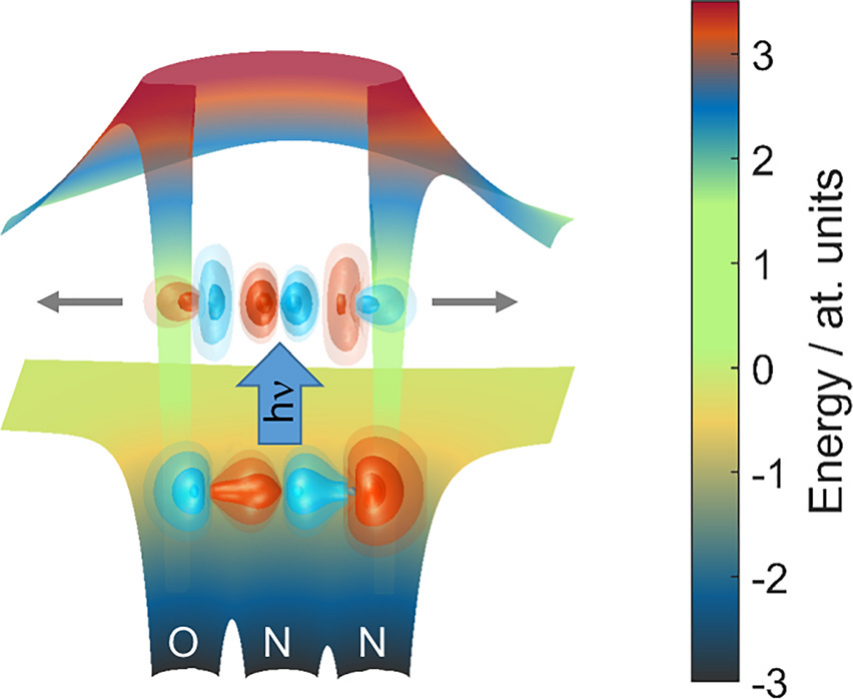
Shape resonance in the case of N_2_O. The lower surface shows the numerically calculated molecular potential containing electrostatic and exchange interactions. The upper surface shows the total potential, i.e., the sum of the molecular and centrifugal potentials. The wave functions of the bound orbital and the shape-resonant state are illustrated by isosurfaces with color-coded signs. The gray arrows represent the tunneling of the photoelectron through the barrier.

Using the RABBITT technique for energy-dependent photoionization delays between the two outermost valence shells of H_2_O and N_2_O in the photon-energy range of 20–40 eV resulted in remarkably large delays of up to 160 as for N_2_O and below 50 as for H_2_O. Comparison with detailed calculations based on the newly developed theory^71,72^ revealed that the large delays in the case of N_2_O are the consequence of a transient trapping of the outgoing photoelectron in shape resonances embedded in the photoionization continua of N_2_O. The calculations indeed predicted a lifetime of ∼110 as for the dominant shape resonance of σ symmetry located in the photon-energy range of interest. These measurements therefore probe the time-domain manifestation of a complex photoemission process consisting of two steps. First, an electron is excited from the initial bound state to a quasi-bound state lying above the ionization threshold. This state, however, has a finite lifetime owing to the presence of a potential barrier created by the superposition of the molecular short-range and the centrifugal potentials. Second, the electron will tunnel through this barrier and escape into the continuum (Fig. 10). In the language of stationary quantum mechanics, the observed delay arises from the rapid variation in the phase of the photoionization matrix elements across the energy domain of the shape resonance. In a time-dependent picture, the delay arises from the trapping of the photoelectron behind the potential barrier.

However, the details of photoionization in the presence of molecular shape resonances are much richer than suggested by this simple interpretation, as can be recognized from the fully differential photoionization delays of N_2_O that are shown and discussed in Ref. 72. The delays in the molecular frame are indeed found to cover a much wider range of many hundred attoseconds, which originates from the structure of the initial orbital, interference effects of multiple partial-wave continuum states, and the structure of the short-range and multipolar potential of the ionic core. Further theoretical work is in progress to gain additional qualitative insights into these results. Importantly, and in contrast to atoms, the effect of the probing infrared field on the delays measured by attosecond interferometry cannot be represented by a simple, additive function, known as the “continuum-continuum delay.”^72^ This difference originates from the non-spherical nature of molecules, and the resulting interference effects in the calculation of the two-photon amplitudes. A better understanding of these effects requires measurements in the molecular frame.

### B. Strong-field ionization rates in polar molecules

The knowledge of strong-field ionization (SFI) rates of molecules is crucial to all attosecond strong-field techniques, including high-harmonic spectroscopy, laser-induced electron diffraction, strong-field photoelectron holography, and the attoclock technique. Strong-field ionization rates are, however, notoriously difficult to calculate because of the non-perturbative nature of the light–matter interaction. Whereas the description of SFI rates of non-polar molecules can be considered to be understood to a reasonable level, polar molecules posed considerable challenges until recently. The main difficulty arises from the presence of a large, strongly angle-dependent Stark shift caused by the permanent dipole moments of the neutral and cationic species. This effect, combined with the exponential sensitivity of the SFI rates to the asymptotic tail of the electronic wave functions undergoing tunnel ionization, called for an innovative theoretical approach and detailed experimental tests thereof.

This challenge has been addressed by the development of the so-called weak-field asymptotic theory (WFAT). The designation “weak field” refers to the electric field as being weak compared with the field strength required for over-barrier ionization. In this sense, intensities of 10^14^ W/cm^2^, typical of attosecond strong-field experiments, are indeed to be considered “weak.” The WFAT has been tested against several experimental results, including high-harmonic spectroscopy^73,74^ and phase-controlled two-color SFI.^75^

In these publications, the WFAT has been combined with all-electron non-perturbative *ab initio* calculations with static applied electric fields to determine the Stark shifts. Specially designed basis sets have been used and their expansion coefficients have been individually optimized to minimize the total electronic energy of the molecule. This procedure led to very accurate asymptotic tails of the molecular orbitals, which yielded highly accurate SFI rates.

The accuracy of these results was validated in several experimental studies, which simultaneously led to new scientific insights. The measurement of high-harmonic spectra of aligned and oriented CH_3_F and CH_3_Br molecules provided the first evidence for the modification of the electronic structure of molecules by the strong electric field driving high-harmonic generation.^73^ Specifically, it was shown that the observed spectra could only be quantitatively explained when the field-modified orbitals were used for calculating the SFI rates and recombination matrix elements and when the additional phase originating from the Stark effect was taken into account. In this case, a quantitative agreement with experimental data was obtained. The validity of the WFAT was further tested in a recent high-harmonic-spectroscopy experiment that reconstructed attosecond charge migration from experimental data.^74^ The angular variation of the SFI rates was required in this work, because of the finite degree of alignment that can be achieved by impulsive methods. The convergence of the experimental retrieval of the time-dependent populations and phases of the electronic eigenstates of the cation support the validity of the SFI rates obtained from the WFAT. The good agreement of the relative ionization rates to the two lowest-lying electronic states of the cation with both a TDDFT calculation and the experiment shows that these quantities are also appropriately predicted by the WFAT. Finally, the predictions of WFAT have also been tested against SFI in a laser pulse consisting of a fundamental frequency and its second harmonic.^75^ The asymmetric emission of fragments from SFI of CH_3_X (with X = F, Cl, Br, I) has been measured, leading to the interesting conclusion that an electron is preferentially removed from the halogen side in CH_3_F, whereas the opposite is the case in the other molecules, with an asymmetry increasing from Cl to I. These results are consistent with the shape of the highest occupied molecular orbitals, but this consideration neglects the role of the Stark shifts which are very important in defining the asymmetry of the SFI rates and are incorporated in the WFAT.

## VI. PHOTOIONIZATION FROM LIQUIDS

In the early 1970s, while developing techniques of photoelectron spectroscopy in the gas phase and at surfaces, Siegbahn and co-workers also introduced photoelectron spectroscopy (PES) of liquids.^76^ However, broader adoption came only in recent years and thanks to the works of Faubel, Winter and their co-workers.^77,78^ It was extended into the ultrafast time domain first in the ultraviolet spectrum (<10 eV),^79–82^ and then the vacuum ultraviolet range.^83,84^ Several groups have since embarked in the implementation of the vacuum ultraviolet (VUV) version of such experiments.^85–87^ The technique has been implemented to investigate the ultrafast IR-induced solvent heating,^83^ intramolecular dynamics of solvated species,^88–91^ and interfacial electron transfer,^92^ while others are exploring extensions into the attosecond regime.^93^

In these implementations of time-resolved PES, the VUV probe field maps the electron distribution of the system under study onto a detector via photoemission, after the pump pulse has perturbed it. When strong fields are used, a dressing of the emitted photoelectrons by the pump laser field can occur.^94^ This leads, among others, to the so-called LAPE (laser-assisted photoelectric effect), when the quasi-monochromatic VUV pulse is longer than the half-cycle duration of the pump field. The manifestation of LAPE is a redistribution of the emitted photoelectron energies into sidebands of the unperturbed spectrum. It was first observed in the gas phase^94,95^ and later from solid surfaces.^96,97^ In recent studies of liquids, the Chergui group has identified LAPE from liquid surfaces of a pure water microjet.^98^

**FIG. 11.**
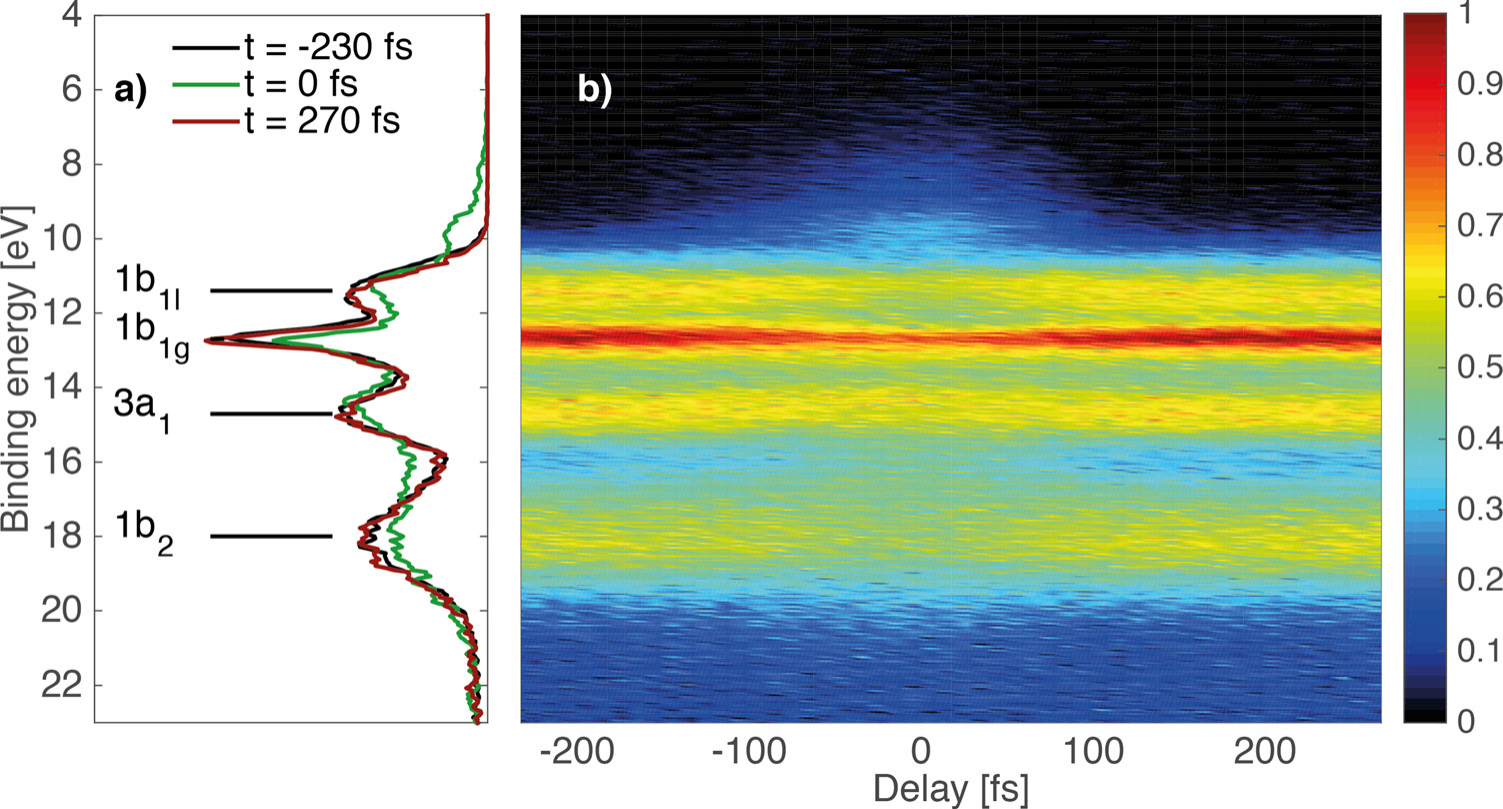
(a) Photoelectron spectrum of water at t = −230 fs (black curve), t = 0 fs (green curve) and t = 270 fs (red curve). The first PE bands of water are shown with assigned molecular orbitals (subscript l refers to the liquid phase, subscript g to the gas phase, no subscript to liquid and gas phase). (b) Evolution of the photoelectron spectrum: Binding energy as a function of laser-pump/XUV probe time delay. Reprinted with permission from Arrell *et al.*, Phys. Rev. Lett. **117**, 143001 (2016).^98^ Copyright 2016 American Physical Society.

**FIG. 12.**
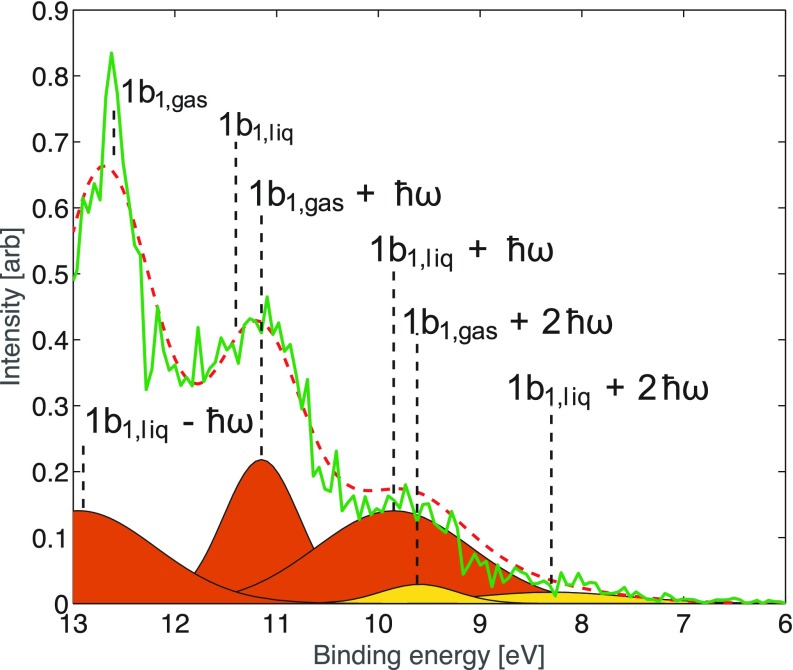
Photoelectron (PE) spectrum of pure water at t = 0 (green trace) showing the sidebands resulting from LAPE and the assignment of the various spectral lines. Sidebands obtained from the Gaussian fit. The first order sidebands are shown in orange and the second order are shown in yellow. Red dashed curve: sum of sidebands and the unperturbed water PE bands. Reprinted with permission from Arrell *et al.*, Phys. Rev. Lett. **117**, 143001 (2016).^98^ Copyright 2016 by American Physical Society.

Figure 11 shows the time evolution of the photoelectron spectra of the pure water jet, and the left panel shows the spectra recorded before, at, and after zero pump-probe delay (t = 0) using a 40 fs pump pulse at 1.55 eV and a probe at 35.6 eV (full width at half maximum = 0.2 eV) of 140 fs duration. The green spectrum at t = 0 clearly shows a redistribution of intensity, in particular below 10 eV, while no signal is detected in this range in the other cases. The detailed assignment of the t = 0 spectrum is shown in Fig. 12. While the main peaks at 11.2 and 12.7 eV represent the binding energy of the 1b_1_ orbital in the gas and the liquid phase, respectively, additional sidebands can easily be identified that are the LAPE lines spaced by integer multiples of the photon energy of the pump laser.

This redistribution of intensity at t = 0 is expected from the interaction of the dressing laser field with the photoemitted electron in a Volkov state of the continuum. This was confirmed by modelling the intensity of the sidebands as described in Ref. 98.

The presence of LAPE in the photoelectron signal around t = 0 offers an *in-situ* optical pump/XUV-probe cross-correlation, which is particularly useful for the study of photoinduced dynamics in a large variety of systems. For experiments at high time resolution, it could be used to study the interfacial effect, as demonstrated for adsorbates on solids by Murnane and co-workers.^96^ We are implementing this technique to measure the core-level relaxation dynamics occurring in solvated species near the water interface.

## VII. PHOTOEMISSION FROM SURFACES

In photoemission from surfaces, photoelectrons excited by light with energies ranging from ultraviolet to x-rays are detected as a function of kinetic energy and emission direction. Due to the small inelastic mean-free path for low-energy electrons in solids, electrons can only escape from a solid from a very thin surface region of nanometer depth without experiencing any inelastic scattering event.^99^ Photoemission spectroscopy is one of the most important methods in surface science and gives access to binding energies and momenta of electrons in initial and final states of the photoexcitation process. While a simple description of the photoemission process in terms of the three steps excitation, transport to the surface, and emission proved useful in many applications, the more accurate quantum mechanical model combines the three steps into a single one (so-called “one-step model”) and automatically includes the surface emission process.^100^

In this model, the final state of the photoexcitation is described as the time-reversal of a free electron impinging from the vacuum on the surface, as in a low-energy electron diffraction (LEED) experiment. For this reason, the final state is commonly called time-reversed LEED state. The LEED electron wavefunction is a plane wave outside the solid. Inside the solid, it may couple to a suitable Bloch state of the same energy and momentum if the respective wavefunctions match at the surface. This case corresponds to the conventional three-step model mentioned above. If no Bloch state is available, the LEED state wavefunction decays exponentially inside the solid on a length scale given by the mean-free path of the electron.^100^ Excitations, which occur within the decay length, lead to additional features in photoelectron spectra, known as surface or gap emission. Actually, it is this process, which is not covered by the three-step model, which makes angle-resolved photoelectron spectroscopy (ARPES) that successful for measuring electron dispersion curves in condensed matter.^99^

Time-resolved ARPES experiments allow one to track the evolution of electronic excitations in surfaces as a function of binding energy and electron momentum.^101^ According to the goal of the experiment, we may, somehow artificially, distinguish two types of experiments: (i) measurements of excited state dynamics and lifetimes and (ii) measurements of time delays related to the photoemission process itself. Due to the very fast timescales of the latter, the experiments always require attosecond time resolution available using special techniques. Both experiments will be elaborated in detail in the corresponding sections in this section. It is anticipated that the distinction between bulk and surface transitions turns out to be crucial for the interpretation of data taken with attosecond resolution.

To this end, it is worth mentioning that static measurements can be used to obtain information about dynamics even on sub-femtosecond timescales. In particular, for the study of charge-transfer dynamics between a substrate and adsorbed molecules, the so-called core-hole clock method proved to be very useful:^102^ briefly, a core electron is excited into an unoccupied state by absorption of an x-ray photon. This excited state can decay by an Auger process, and the energy of the emitted Auger electron depends on the energy levels involved and the screening by the core electron, which was excited into a valence state. The relative intensities of the Auger peaks in the spectra are proportional to the ratio of the electron transfer rate to the core hole decay rate.^103^ If the core hole lifetime τ is known from high-resolution spectroscopy, electron transfer times between 0.1 τ and 10 τ, thus about 0.5–50 fs, can be measured with very high precision from the intensities of the respective Auger peaks.^104^

### A. Two-photon photoemission

**FIG. 13.**
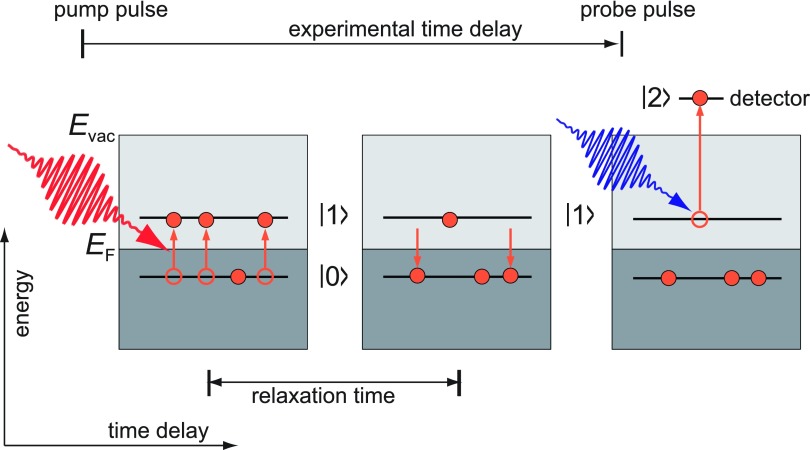
Sketch of the 2PPE experiment. E_F_ refers to the Fermi energy of the solid. Electrons are excited from an initial state |0⟩ into an intermediate state |1⟩ by absorption of photons from the pump pulse. The intermediate state decays on a typical relaxation time. Absorption of a second photon from the probe pulse promotes electrons from the intermediate state to the final state |2⟩. The final signal measured in the detector is proportional to the final state population.

In the two-photon photoemission (2PPE) experiment, an electron is excited and emitted in a two-step process requiring two photons with the photon energies of each of them individually not being sufficient to lead to photoemission. The process is schematically shown in Fig. 13. 2PPE proved to be an excellent tool for the investigation of unoccupied electronic states at metal and semiconductor surfaces and in molecular layers, and numerous examples can be found in the reviews^105^ and^106^ for instance.

The recorded signal is proportional to the population of the final state |2⟩. For the analysis of single transients, the solution of rate equations is sufficient. A full description of transition rates and energy spectra can be obtained within the density matrix formalism: The differential equations are named optical Bloch equations.^107^ Using rotating wave approximation to replace the high frequency fields by DC fields, the equations can be solved analytically allowing the energy spectra to be calculated.^108,109^ The final spectra can be expressed in terms of transition rates and decay rates. As a result, the transients are dominated by population decay rates, and the spectral linewidth is dictated by population decay *and* dephasing rates. The latter are of the order of a few femtoseconds in ordinary metals and thus dominate the linewidths. The coherence, however, built up between initial and intermediate states in the first excitation step leads to quantum beat phenomena^110^ and can be used to induce surface currents by selective excitation of electrons at certain momenta in reciprocal space.^111^

Here, we want to focus on the emission mechanism from surfaces with a negative electron affinity. Diamondoid molecules, which are small hydrocarbon molecules with a diamond structure, grow in self-assembled monolayers on noble-metal surfaces.^112^ For the case of^121^-tetramantane-thiol on Ag(111), Yang and co-workers could show using synchrotron radiation that the photoemission spectrum is dominated by a very strong peak slightly above the vacuum level. The energy position is independent of photon energy and corresponds to the energy position of the lowest unoccupied molecular orbital (LUMO) of the diamondoid,^112^ giving strong evidence for the negative electron affinity of the diamondoid layer. Negative electron affinity means that the LUMO energy is higher than the vacuum energy. Any electron promoted into the LUMO can be spontaneously emitted.^113^ In order to elucidate the excitation mechanism, 2PPE was used to determine the unoccupied electronic structure and the lifetimes and ionization delays.

**FIG. 14.**
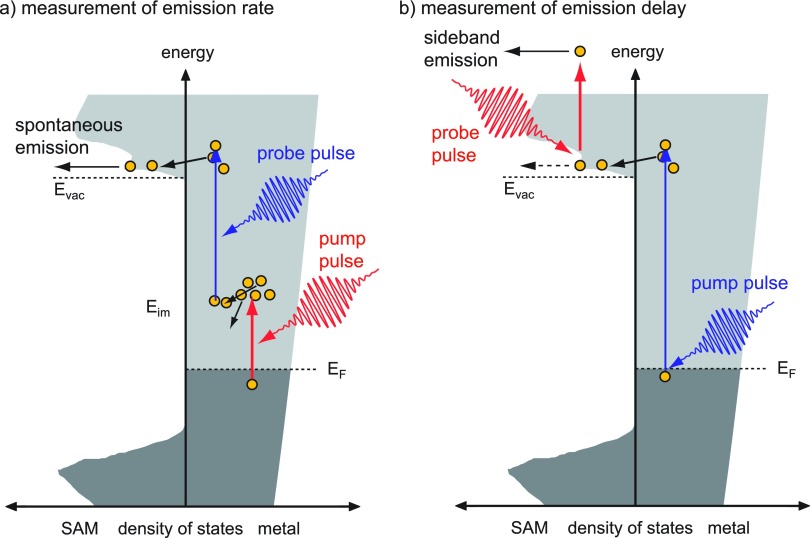
2PPE from negative-electron affinity layers with two possible pulse sequences: (a) generation of a hot electron gas in the substrate followed by a second pulse exciting the electrons to levels above the LUMO of the diamondoid. An efficient electron transfer mechanism into the LUMO and spontaneous emission from the LUMO lead to an intense and monochromatic electron spectrum.^112^ In such experiments, the emission rate is measured as a function of pump-probe delay. (b) If one of the pulses is sufficient to generate photoelectrons, the second pulse can be used to clock the emission. In this case, the electron may absorb a photon during its passage through the LUMO. In the spectrum, the electron would be detected as side band shifted by the probe photon energy from the main peak. Adapted from Roth *et al.*, Chem. Phys. Lett. **495**, 102–108 (2010). Copyright 2010 Elsevier.^114^

Two types of experiments were carried out: first, the emission rate from the LUMO was measured as a function of pump-probe delay [Fig. 14(a)]. It could be shown that the electrons are mostly excited in the metallic substrate and transferred to the molecule; the transient showed typical exponential decay with lifetimes in agreement with Fermi liquid theory models for silver.^114^

In a second experiment, the emission delay was to be measured, i.e., the time between the initial excitation pulse and the moment when the electron leaves the surface. For this experiment, the sequence was reverted using an intense infrared pulse as probe. If the electron is in the LUMO when the probe pulse is present, the electron can absorb a photon and appear in a sideband at a higher kinetic energy. This effect is called LAPE (laser-assisted photoelectric effect)^96^ (see also Sec. VI). At a first glance, it seems to be similar to the RABBITT technique, which was discussed for the gas phase and which will be presented in the context of photoemission from solids in Sec. VII B, below. However, the LAPE concept is different because it only involves a single XUV photon energy. The sideband yield gives information about the probability to find an electron in the LUMO.^115^ As a result, an upper bound of a few femtoseconds could be determined for the retention time in the LUMO.^114^ This result is in agreement with results from tunneling experiments from negative-affinity Ar layers adsorbed on a Cu surface.^116^

### B. Surface RABBITT

While typical 2PPE experiments probe delays in the range of tens of femtoseconds to picoseconds, even smaller photoemission delays on an attosecond timescale can be resolved in dedicated experiments. In the first attosecond streaking experiment in condensed matter, Cavalieri *et al.* found small relative delays between the valence band and core level photoemission from W(110) on the order of 100 as.^117^ Relative delays in the same range were found in subsequent streaking experiments from Mg(0001)^118,119^ and Au and WO_3._^120^

The measured relative delay of 110 as between *4f* and conduction band electrons in the first attosecond experiment in condensed matter gave rise to a considerable amount of theoretical investigations. The differing emission times were explained in terms of different initial state localization,^121,122^ penetration of the surface barrier,^123^ resonant transitions,^124^ and electron transport.^121,125^ In analogy to atomic photoionization, a Wigner delay in photoemission from solid surfaces has been discussed as the consequence of an accumulated phase shift of the propagating wave packet^126^ as well as the result of inherent phase-shifts associated with final state effects in photoemission.^127,128^

**FIG. 15.**
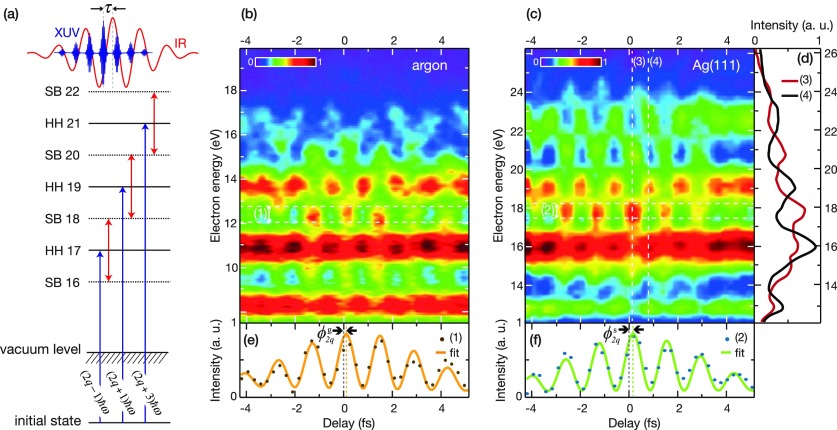
(a) Energy level scheme of the RABBITT process. Interfering two-color two-photon transitions give rise to sidebands (SB) between adjacent odd high harmonics (HH). (b) and (c) Experimental RABBITT traces from Ar and Ag(111) with electrons originating from Ar 3p and Ag 4d levels, respectively. Both scans were recorded simultaneously with laser parameters optimized for the surface. A delay-independent background of ATP and secondary electrons was subtracted from (c) to enhance contrast for illustration purposes. (d) Photoelectron spectra from (c) at two different delays. At 100 as (3), the appearance of sidebands is clearly visible, whereas at 800 as (4), the photoelectron spectrum qualitatively resembles the spectrum in the absence of the IR field. (e) and (f) Integration over the energy range of SB 18 revealing the oscillation with 2ω. Experimental curves (1) and (2) were fitted with A(t) cos(2ωt − ϕ_2q_) where ϕ_2q_ is the experimental spectral phase as indicated and A(t) is the pulse envelope function. Reprinted with permission from Locher *et al.*, Optica **2**, 405–410 (2015).^115^ Copyright 2015 Optical Society of America.

While the first attosecond experiments on solid surfaces were based on attosecond streaking,^17^ the later application of RABBITT^18,19^ had several decisive advantages: (i) the required intensity of the IR probe field is significantly lower, thereby leading to less perturbation of the studied system as well as reduced above-threshold photoemission (ATP) background. (ii) RABBITT intrinsically yields energy resolution through its discrete sidebands, while energy resolution in streaking is only obtained through applying suitable reconstruction algorithms. For these two reasons, RABBITT is an ideal tool to study the photoemission dynamics of valence states at relatively low excitation energies. A first surface RABBITT experiment^115^ found a strong energy-dependent variation of photoemission delays from the d-valence bands of Ag(111) and Au(111). This experiment took advantage of a unique experimental geometry^28^ that allowed for simultaneous RABBITT measurements in a gas phase target and on a solid surface target. Reference measurements in Ar allowed for on-the-fly calibration of the harmonic phase and in principle enable the determination of absolute photoemission delays as shown in Fig. 15.

**FIG. 16.**
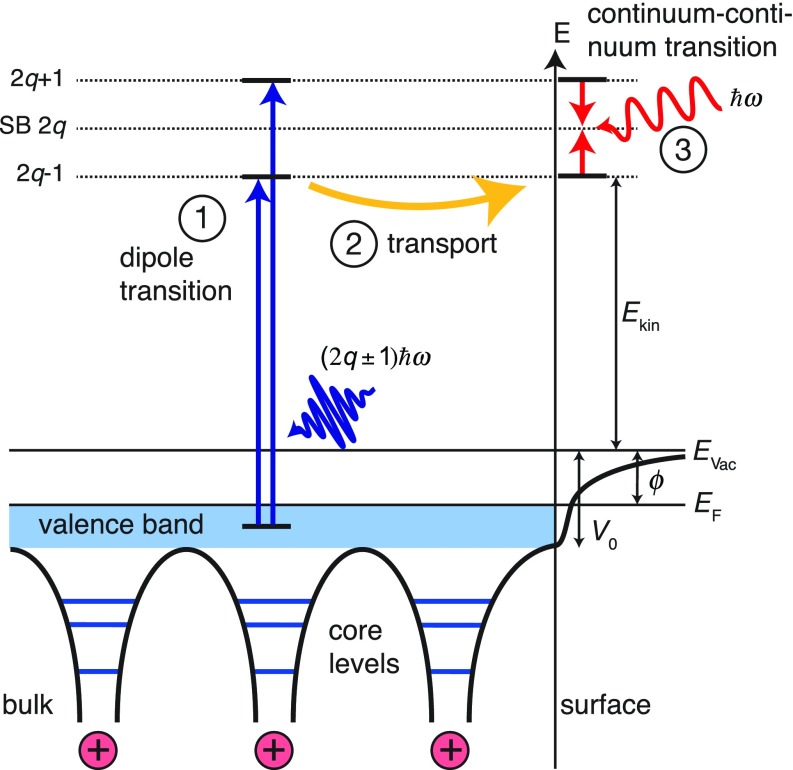
Schematic representation of the three steps involved in the surface RABBITT: (1) initial excitation of the electron by absorption of an XUV photon, (2) transport within the solid, and (3) absorption/emission of an IR photon.

In the measurements of photoemission of Ag(111) and Au(111), all detected electrons originated from the same initial states, namely the 4d and 5d valence bands, respectively. Initial state effects such as different localization could thus be ruled out as the origin of the observed variation of the delays. Model calculations were carried out based on a simple three-step model consisting of initial (1) photoexcitation, (2) transport, and (3) interaction with the IR probe field (see Fig. 16). The results showed that both the initial XUV excitation as well as the IR-induced continuum-continuum transition contributed relatively little to the total Wigner delay compared with the transport times. This observation was further supported very recently in a RABBITT study from Ni(111).^129^ Moreover, given the very high reflectivity in the IR of the investigated noble metal surfaces, it was assumed that the IR field was screened very efficiently, and an interaction of the outgoing photoelectron wavepacket with the probe field took place at the surface.^115^ This assumption was later confirmed in an experiment on Cu(111) where the phase of the IR induced transient-grating was determined for different incidence angles of the light.^130^ It could be demonstrated that the macroscopic Fresnel laws even hold on atomic length and time scales. RABBITT was performed on Cu(111) with IR incidence angles of 15° and 75°, respectively. The sample was rotated in a way that the same momentum space and thus same initial states were probed for both incidence angles. Any difference in the measured photoemission phase must thus be related to the phase of the probe field. Since the measured phase difference was in agreement with the Fresnel calculations for the reflecting case, it could further be concluded that the IR field does not penetrate the solid.

## VIII. CONCLUSION AND OUTLOOK

This review has given an overview of the current state-of-the-art experiments studying the dynamical aspects of ionization and photoemission in a wide range of physical systems—from simple atoms to molecules, liquids and solids. This research has only become possible with the rapid progress in experimental tools for time-resolved studies. Ionization and photoemission are processes of fundamental importance in nature and technology. This overview also shows that many questions still remain open in this field. On the one hand, as we approach more complex systems—as, for example, those being representative for real biological systems—dynamics can become very complicated and it is a formidable challenge to disentangle all relevant mechanisms. On the other hand, the exciting experimental possibilities offered by attosecond science force us to reconsider our understanding and interpretation of fundamental quantum mechanics. It is therefore expected that the wider field of ionization and photoemission remains a hot area of active research for many years to come.

## NOMENCLATURE

2PPE: Two-photo photoemission
APT: Attosecond pulse train
ARPES: Angle-resolved photoelectron spectroscopy
ATP: Above-threshold photoemission
COLTRIMS: Cold target recoil ion momentum spectroscopy
CPMD: Car-Parrinello molecular dynamics
FPI: Feynman path integral
FROG-CRAB: Frequency-resolved optical gating for the complete reconstruction of attosecond bursts
IR: Infrared
LAPE: Laser-assisted photoelectric effect
LEED: Low-energy electron diffraction
LUMO: Lowest unoccupied molecular orbital
PBE: Perdew, Burke, Ernzerhof
PES: Photoelectron spectroscopy
RABBITT: Reconstruction of attosecond beating by the interference of two-photon transitions
SAE: Single active electron approximation
SB: Sidebands
SFI: Strong-field ionization
TD-CIS: Time-dependent configuration-interaction singles
TDDFT: Time-dependent density functional theory
TDSE: Time-dependent Schrödinger equation
TIPIS: Tunnel ionization in parabolic coordinates with induced dipole and Stark-shift
VUV: Vacuum ultraviolet
WFAT: Weak-field asymptotic theory
XUV: Extreme ultraviolet

## References

[1] C. Nordling , E. Sokolowski , and K. Siegbahn , “ Precision method for obtaining absolute values of atomic binding energies,” Phys. Rev. 105, 1676–1677 (1957). 10.1103/PhysRev.105.1676

[2] D. W. Turner and M. I Al Jobory , “ Determination of ionization potentials by photoelectron energy measurement,” J. Chem. Phys. 37, 3007 (1962). 10.1063/1.1733134

[3] T. Fauster and W. Steinmann , “ Two-photon photoemission spectroscopy of image states,” in *Electromagnetic Waves: Recent Developments in Research*, edited by Halevi P. ( Elsevier, Amsterdam, 1995), pp. 347–411.

[4] H. Hertz , “ Ueber einen Einfluss des ultravioletten Lichtes auf die electrische Entladung” (“About an influence of ultraviolet light on the electric discharge”), Ann. Phys. 267, 983–1000 (1887). 10.1002/andp.18872670827

[5] A. Einstein , “ Ueber einen die Erzeugung und Verwandlung des Lichtes betreffenden heuristischen Gesichtspunkt” (“On a heuristic point of view concerning the production and transformation of light”), Ann. Phys. 322, 132–148 (1905). 10.1002/andp.19053220607

[6] A. Ludwig , J. Maurer , B. W. Mayer , C. R. Phillips , L. Gallmann , and U. Keller , “ Breakdown of the dipole approximation in strong-field ionization,” Phys. Rev. Lett. 113, 243001 (2014). 10.1103/PhysRevLett.113.243001 25541770

[7] H. R. Reiss , “ The tunnelling model of laser-induced ionization and its failure at low frequencies,” J. Phys. B: At. Mol. Opt. Phys. 47, 204006 (2014). 10.1088/0953-4075/47/20/204006

[8] L. V. Keldysh , “ Ionization in the field of a strong electromagnetic wave,” Sov. Phys. JETP 20, 1307–1314 (1965), available at http://www.jetp.ac.ru/cgi-bin/e/index/e/20/5/p1307?a=list.

[9] E. P. Wigner , “ Lower limit for the energy derivative of the scattering phase shift,” Phys. Rev. 98, 145–147 (1955). 10.1103/PhysRev.98.145

[10] F. T. Smith , “ Lifetime matrix in collision theory,” Phys. Rev. 118, 349–356 (1960). 10.1103/PhysRev.118.349

[11] A. S. Landsman , M. Weger , J. Maurer , R. Boge , A. Ludwig , S. Heuser , C. Cirelli , L. Gallmann , and U. Keller , “ Ultrafast resolution of tunneling delay time,” Optica 1, 343–349 (2014). 10.1364/OPTICA.1.000343

[12] A. S. Landsman and U. Keller , “ Attosecond science and the tunneling time problem,” Phys. Rep. 547, 1–24 (2015). 10.1016/j.physrep.2014.09.002

[13] P. Eckle , A. N. Pfeiffer , C. Cirelli , A. Staudte , R. Dörner , H. G. Muller , M. Büttiker , and U. Keller , “ Attosecond ionization and tunneling delay time measurements in helium,” Science 322, 1525–1529 (2008). 10.1126/science.1163439 19056981

[14] L. Torlina , F. Morales , J. Kaushal , I. Ivanov , A. Kheifets , A. Zielinski , A. Scrinzi , H. G. Muller , S. Sukiasyan , M. Ivanov , and O. Smirnova , “ Interpreting attoclock measurements of tunnelling times,” Nat. Phys. 11, 503–508 (2015). 10.1038/nphys3340

[15] V. P. Majety and A. Scrinzi , “ Absence of electron correlation effects in the Helium attoclock setting,” J. Mod. Opt. 64, 1026–1030 (2017). 10.1080/09500340.2016.1271915

[16] M. Hentschel , R. Kienberger , C. Spielmann , G. A. Reider , N. Milosevic , T. Brabec , P. Corkum , U. Heinzmann , M. Drescher , and F. Krausz , “ Attosecond metrology,” Nature 414, 509–513 (2001). 10.1038/35107000 11734845

[17] J. Itatani , F. Quéré , G. L. Yudin , M. Y. Ivanov , F. Krausz , and P. B. Corkum , “ Attosecond streak camera,” Phys. Rev. Lett. 88, 173903 (2002). 10.1103/PhysRevLett.88.173903 12005756

[18] H. G. Muller , “ Reconstruction of attosecond harmonic beating by interference of two-photon transitions,” Appl. Phys. B 74, S17–S21 (2002). 10.1007/s00340-002-0894-8

[19] P. M. Paul , E. S. Toma , P. Breger , G. Mullot , F. Augé , P. Balcou , H. G. Muller , and P. Agostini , “ Observation of a train of attosecond pulses from high harmonic generation,” Science 292, 1689–1692 (2001). 10.1126/science.1059413 11387467

[20] R. Kienberger , E. Goulielmakis , M. Uiberacker , A. Baltuska , V. Yakovlev , U. Heinzmann , M. Drescher , and F. Krausz , “ Atomic transient recorder,” Nature 427, 817–821 (2004). 10.1038/nature02277 14985755

[21] E. Goulielmakis , M. Schultze , M. Hofstetter , V. S. Yakovlev , J. Gagnon , M. Uiberacker , A. L. Aquila , E. M. Gullikson , D. T. Attwood , R. Kienberger , F. Krausz , and U. Kleineberg , “ Single-cycle nonlinear optics,” Science 320, 1614–1617 (2008). 10.1126/science.1157846 18566281

[22] K. Klünder , J. M. Dahlström , M. Gisselbrecht , T. Fordell , M. Swoboda , D. Guenot , P. Johnsson , J. Caillat , J. Mauritsson , A. Maquet , R. Taïeb , and A. L'Huillier , “ Probing single-photon ionization on the attosecond time scale,” Phys. Rev. Lett. 106, 143002 (2011). 10.1103/PhysRevLett.106.143002 21561188

[23] M. Schultze , M. Fiess , N. Karpowicz , J. Gagnon , M. Korbman , M. Hofstetter , S. Neppl , A. L. Cavalieri , Y. Komninos , T. Mercouris , C. A. Nicolaides , R. Pazourek , S. Nagele , J. Feist , J. Burgdorfer , A. M. Azzeer , R. Ernstorfer , R. Kienberger , U. Kleineberg , E. Goulielmakis , F. Krausz , and V. S. Yakovlev , “ Delay in photoemission,” Science 328, 1658–1662 (2010). 10.1126/science.1189401 20576884

[24] M. Sabbar , S. Heuser , R. Boge , M. Lucchini , L. Gallmann , C. Cirelli , and U. Keller , “ Combining attosecond XUV pulses with coincidence spectroscopy,” Rev. Sci. Instrum. 85, 103113 (2014). 10.1063/1.4898017 25362377

[25] M. Sabbar , S. Heuser , R. Boge , M. Lucchini , T. Carette , E. Lindroth , L. Gallmann , C. Cirelli , and U. Keller , “ Resonance effects in photoemission time delays,” Phys. Rev. Lett. 115, 133001 (2015). 10.1103/PhysRevLett.115.133001 26451550

[26] L. Cattaneo , J. Vos , M. Lucchini , L. Gallmann , C. Cirelli , and U. Keller , “ Comparison of attosecond streaking and RABBITT,” Opt. Express 24, 29060 (2016). 10.1364/OE.24.029060 27958571

[27] J. M. Dahlström , A. L'Huillier , and A. Maquet , “ Introduction to attosecond delays in photoionization,” J. Phys. B: At. Mol. Opt. Phys. 45, 183001 (2012). 10.1088/0953-4075/45/18/183001

[28] R. Locher , M. Lucchini , J. Herrmann , M. Sabbar , M. Weger , L. A , L. Castiglioni , M. Greif , M. Hengsberger , L. Gallmann , and U. Keller , “ Versatile attosecond beamline in a two-foci configuration for simultaneous time-resolved measurements,” Rev. Sci. Instrum. 85, 013113 (2014). 10.1063/1.4862656 24517751

[29] J. M. Dahlström , D. Guénot , K. Klünder , M. Gisselbrecht , J. Mauritsson , A. L'Huillier , A. Maquet , and R. Taïeb , “ Theory of attosecond delays in laser-assisted photoionization,” Chem. Phys. 414, 53–64 (2013). 10.1016/j.chemphys.2012.01.017

[30] M. Kitzler , N. Milosevic , A. Scrinzi , F. Krausz , and T. Brabec , “ Quantum theory of attosecond XUV pulse measurement by laser-dressed photoionization,” Phys. Rev. Lett. 88, 173904 (2002). 10.1103/PhysRevLett.88.173904 12005757

[31] R. Kienberger , M. Hentschel , M. Uiberacker , C. Spielmann , M. Kitzler , A. Scrinzi , M. Wieland , T. Westerwalbesloh , U. Kleineberg , U. Heinzmann , M. Drescher , and F. Krausz , “ Steering attosecond electron wave packets with light,” Science 297, 1144–1148 (2002). 10.1126/science.1073866 12114530

[32] Y. Mairesse and F. Quéré , “ Frequency-resolved optical gating for complete reconstruction of attosecond bursts,” Phys. Rev. A 71, 011401 (2005). 10.1103/PhysRevA.71.011401

[33] J. Gagnon and V. S. Yakovlev , “ The robustness of attosecond streaking measurements,” Opt. Express 17, 17678–17693 (2009). 10.1364/OE.17.017678 19907553

[34] R. Pazourek , S. Nagele , and J. Burgdörfer , “ Time-resolved photoemission on the attosecond scale: Opportunities and challenges,” Faraday Discuss. 163, 353–376 (2013). 10.1039/c3fd00004d 24020211

[35] M. Lucchini , A. Ludwig , L. Kasmi , L. Gallmann , and U. Keller , “ Semi-classical approach to compute RABBITT traces in multi-dimensional complex field distributions,” Opt. Express 23, 8867–8879 (2015). 10.1364/OE.23.008867 25968724

[36] R. Pazourek , S. Nagele , and J. Burgdörfer , “ Attosecond chronoscopy of photoemission,” Rev. Mod. Phys. 87, 765–802 (2015). 10.1103/RevModPhys.87.765

[37] R. Dörner , V. Mergel , O. Jagutzki , L. Spielberger , J. Ullrich , R. Moshammer , and H. Schmidt-Bocking , “ Cold target recoil ion momentum spectroscopy: A ‘momentum microscope’ to view atomic collision dynamics,” Phys. Rep. 330, 95–192 (2000). 10.1016/S0370-1573(99)00109-X

[38] A. S. Kheifets , “ Time delay in valence-shell photoionization of noble-gas atoms,” Phys. Rev. A 87, 063404 (2013). 10.1103/PhysRevA.87.063404

[39] T. Carette , J. M. Dahlström , L. Argenti , and E. Lindroth , “ Multiconfigurational Hartree-Fock close-coupling ansatz: Application to the argon photoionization cross section and delays,” Phys. Rev. A 87, 023420 (2013). 10.1103/PhysRevA.87.023420

[40] M. Kotur , D. Guénot , Á. Jiménez-Galán , D. Kroon , E. W. Larsen , M. Louisy , S. Bengtsson , M. Miranda , J. Mauritsson , C. L. Arnold , S. E. Canton , M. Gisselbrecht , T. Carette , J. M. Dahlström , E. Lindroth , A. Maquet , L. Argenti , F. Martín , and A. L'Huillier , “ Spectral phase measurement of a Fano resonance using tunable attosecond pulses,” Nat. Commun. 7, 10566 (2016). 10.1038/ncomms10566 26887682 PMC4759632

[41] A. Jiménez-Galán , L. Argenti , and F. Martin , “ Modulation of attosecond beating in resonant two-photon ionization,” Phys. Rev. Lett. 113, 263001 (2014). 10.1103/PhysRevLett.113.263001 25615319

[42] J. M. Dahlström and E. Lindroth , “ Study of attosecond delays using perturbation diagrams and exterior complex scaling,” J. Phys. B: At., Mol. Opt. Phys. 47, 124012 (2014). 10.1088/0953-4075/47/12/124012

[43] S. Heuser , Á. Jiménez-Galán , C. Cirelli , M. Sabbar , R. Boge , M. Lucchini , L. Gallmann , I. Ivanov , A. S. Kheifets , J. M. Dahlström , E. Lindroth , L. Argenti , F. Martín , and U. Keller , “ Angular dependence of photoemission time delay in helium,” Phys. Rev. A 94, 063409 (2016). 10.1103/PhysRevA.94.063409

[44] I. Jordan , M. Huppert , S. Pabst , A. Kheifets , D. Baykusheva , and H. J. Wörner , “ Spin-orbit delays in photoemission,” Phys. Rev. A 95, 013404 (2017). 10.1103/PhysRevA.95.013404

[45] M. Baggash and H. Rottke , “ Phase differences in the photoemission from krypton in the fine-structure-split ionization channels 2P3/2 and 2P1/2,” Phys. Rev. A 92, 013424 (2015). 10.1103/PhysRevA.92.013424

[46] L. Greenman , P. J. Ho , S. Pabst , E. Kamarchik , D. A. Mazziotti , and R. Santra , “ Implementation of the time-dependent configuration-interaction singles method for atomic strong-field processes,” Phys. Rev. A 82, 023406 (2010). 10.1103/PhysRevA.82.023406

[47] A. Enders and G. Nimtz , “ On superluminal barrier traversal,” J. Phys. I France 2, 1693 (1992). 10.1051/jp1:1992236

[48] C. Cirelli , C. Marante , S. Heuser , C. L. M. Petersson , Á. J. Galán , L. Argenti , S. Zhong , D. Busto , M. Isinger , S. Nandi , S. Maclot , L. Rading , P. Johnsson , M. Gisselbrecht , M. Lucchini , L. Gallmann , J. M. Dahlström , E. Lindroth , A. L'Huillier , F. Martín , and U. Keller , “ Anisotropic photoemission time delays close to a Fano resonance” (unpublished). 10.1038/s41467-018-03009-1 PMC5840338 29511164

[49] A. M. Perelomov , V. S. Popov , and M. V. Terentev , “ Ionization of atoms in an alternating electric field,” Sov. Phys. JETP 50, 1393 (1966), available at http://www.jetp.ac.ru/cgi-bin/e/index/e/23/5/p924?a=list.

[50] P. Eckle , M. Smolarski , P. Schlup , J. Biegert , A. Staudte , M. Schöffler , H. G. Muller , R. Dörner , and U. Keller , “ Attosecond angular streaking,” Nat. Phys. 4, 565–570 (2008). 10.1038/nphys982

[51] A. N. Pfeiffer , C. Cirelli , M. Smolarski , D. Dimitrovski , M. Abu-samha , L. B. Madsen , and U. Keller , “ Attoclock reveals natural coordinates of the laser-induced tunnelling current flow in atoms,” Nat. Phys. 8, 76 (2012). 10.1038/nphys2125

[52] A. N. Pfeiffer , C. Cirelli , M. Smolarski , R. Dorner , and U. Keller , “ Timing the release in sequential double ionization,” Nat. Phys. 7, 428–433 (2011). 10.1038/nphys1946

[53] R. Boge , C. Cirelli , A. S. Landsman , S. Heuser , A. Ludwig , J. Maurer , M. Weger , L. Gallmann , and U. Keller , “ Probing non-adiabatic effects in strong-field tunnel ionization,” Phys. Rev. Lett. 111, 103003 (2013). 10.1103/PhysRevLett.111.103003 25166662

[54] I. Dreissigacker and M. Lein , “ Quantitative theory for the lateral momentum distribution after strong-field ionization,” Chem. Phys. 414, 69–72 (2013). 10.1016/j.chemphys.2012.01.028

[55] C. Hofmann , A. S. Landsman , A. Zielinski , C. Cirelli , T. Zimmermann , A. Scrinzi , and U. Keller , “ Interpreting electron-momentum distributions and nonadiabaticity in strong-field ionization,” Phys. Rev. A 90, 043406 (2014). 10.1103/PhysRevA.90.043406

[56] C. Hofmann , T. Zimmermann , A. Zielinski , and A. S. Landsman , “ Non-adiabatic imprints on the electron wave packet in strong field ionization with circular polarization,” New J. Phys. 18, 043011 (2016). 10.1088/1367-2630/18/4/043011

[57] C. Hofmann , A. S. Landman , and U. Keller , “Attoclock revisited” (unpublished).

[58] A. Emmanouilidou , A. Chen , C. Hofmann , U. Keller , and A. S. Landsman , “ The effect of electron-electron correlation on the attoclock experiment,” J. Phys. B: At. Mol. Opt. Phys. 48, 245602 (2015). 10.1088/0953-4075/48/24/245602

[59] A. Castro , M. A. L. Marques , and A. Rubio , “ Propagators for the time-dependent Kohn-Sham equations,” J. Chem. Phys. 121, 3425–3433 (2004). 10.1063/1.1774980 15303905

[60] Copyright MPI für Festkörperforschung S.-. Copyright IBM Corp, “CPMD V3.15.3,” (1990–2008).

[61] J. P. Perdew , K. Burke , and M. Ernzerhof , “ Generalized gradient approximation made simple,” Phys. Rev. Lett. 77, 3865–3868 (1996). 10.1103/PhysRevLett.77.3865 10062328

[62] R. van Leeuwen and E. J. Baerends , “ Exchange-correlation potential with correct asymptotic behavior,” Phys. Rev. A 49, 2421 (1994). 10.1103/PhysRevA.49.2421 9910514

[63] N. Troullier and J. L. Martins , “ Efficient pseudopotentials for plane-wave calculations,” Phys. Rev. B 43, 1993 (1991). 10.1103/PhysRevB.43.1993 9997467

[64] C. A. Ullrich , “ Time-dependent Kohn-Sham approach to multiple ionization,” J. Mol. Struct.: Theochem. 501, 315–325 (2000). 10.1016/S0166-1280(99)00442-X

[65] I. Barth and O. Smirnova , “ Nonadiabatic tunneling in circularly polarized laser fields: Physical picture and calculations,” Phys. Rev. A 84, 063415 (2011). 10.1103/PhysRevA.84.063415

[66] A. N. Pfeiffer , C. Cirelli , M. Smolarski , and U. Keller , “ Recent attoclock measurements of strong field ionization,” Chem. Phys. 414, 84–91 (2013). 10.1016/j.chemphys.2012.02.005

[67] T. Herath , L. Yan , S. K. Lee , and W. Li , “ Strong-field ionization rate depends on the sign of the magnetic quantum number,” Phys. Rev. Lett. 109, 043004 (2012). 10.1103/PhysRevLett.109.043004 23006084

[68] X. M. Tong and C. D. Lin , “ Empirical formula for static field ionization rates of atoms and molecules by lasers in the barrier-suppression regime,” J. Phys. B 38, 2593–2600 (2005). 10.1088/0953-4075/38/15/001

[69] S. Heuser , C. Cirelli , M. Sabbar , R. Boge , M. Lucchini , L. Gallmann , and U. Keller , “ Influence of autoionizing states on the photoionization dynamics of H2,” in *Ultrafast Dynamic Imaging of Matter (UDIM)* ( Grindelwald, Switzerland, 2015).

[70] L. Cattaneo , J. Vos , S. Heuser , M. Lucchini , C. Cirelli , and U. Keller , “ Asymmetric Wigner time delay in CO photoionization,” in *Ultrafast Phenomena* ( Santa Fe, NM, 2016), p. UM2B.3.

[71] M. Huppert , I. Jordan , D. Baykusheva , A. v. Conta , and H. J. Wörner , “ Attosecond delays in molecular photoionization,” Phys. Rev. Lett. 117, 093001 (2016). 10.1103/PhysRevLett.117.093001 27610849

[72] D. Baykusheva and H. J. Wörner , “ Theory of attosecond delays in molecular photoionization,” J. Chem. Phys. 146, 124306 (2017). 10.1063/1.4977933 28388142

[73] P. M. Kraus , O. I. Tolstikhin , D. Baykusheva , A. Rupenyan , J. Schneider , C. Z. Bisgaard , T. Morishita , F. Jensen , L. B. Madsen , and H. J. Wörner , “ Observation of laser-induced electronic structure in oriented polyatomic molecules,” Nat. Commun. 6, 7039 (2015). 10.1038/ncomms8039 25940229 PMC4432593

[74] P. M. Kraus , B. Mignolet , D. Baykusheva , A. Rupenyan , L. Horný , E. F. Penka , G. Grassi , O. I. Tolstikhin , J. Schneider , F. Jensen , L. B. Madsen , A. D. Bandrauk , F. Remacle , and H. J. Wörner , “ Measurement and laser control of attosecond charge migration in ionized iodoacetylene,” Science 350, 790–795 (2015). 10.1126/science.aab2160 26494175

[75] S. G. Walt , N. Bhargava Ram , A. von Conta , O. I. Tolstikhin , L. B. Madsen , F. Jensen , and H. J. Wörner , “ Role of multi-electron effects in the asymmetry of strong-field ionization and fragmentation of polar molecules: The Methyl halide series,” J. Phys. Chem. 119, 11772–11782 (2015). 10.1021/acs.jpca.5b07331 26565126

[76] H. Siegbahn and K. Siegbahn , “ ESCA applied to liquids,” J. Electron. Spectrosc. Relat. Phenom. 2, 319–325 (1973). 10.1016/0368-2048(73)80023-4

[77] M. Faubel , B. Steiner , and J. P. Toennies , “ Photoelectron spectroscopy of liquid water, some alcohols, and pure nonane in free micro jets,” J. Chem. Phys. 106, 9013–9031 (1997). 10.1063/1.474034

[78] B. Winter , R. Weber , W. Widdra , M. Dittmar , M. Faubel , and I. V. Hertel , “ Full valence band photoemission from liquid water using EUV synchrotron radiation,” J. Phys. Chem. A 108, 2625–2632 (2004). 10.1021/jp030263q

[79] Y. Tang , H. Shen , K. Sekiguchi , N. Kurahashi , T. Mizuno , Y.-I. Suzuki , and T. Suzuki , “ Direct measurement of vertical binding energy of a hydrated electron,” Phys. Chem. Chem. Phys. 12, 3653–3655 (2010). 10.1039/b925741a 20358061

[80] A. T. Shreve , T. A. Yen , and D. M. Neumark , “ Photoelectron spectroscopy of hydrated electrons,” Chem. Phys. Lett. 493, 216–219 (2010). 10.1016/j.cplett.2010.05.059

[81] A. Lübcke , F. Buchner , N. Heine , I. V. Hertel , and T. Schultz , “ Time-resolved photoelectron spectroscopy of solvated electrons in aqueous NaI solution,” Phys. Chem. Chem. Phys. 12, 14629–14634 (2010). 10.1039/c0cp00847h 20886131

[82] F. Buchner , B. Heggen , H.-H. Ritze , W. Thiel , and A. Lübcke , “ Excited-state dynamics of guanosine in aqueous solution revealed by time-resolved photoelectron spectroscopy: Experiment and theory,” Phys. Chem. Chem. Phys. 17, 31978–31987 (2015). 10.1039/C5CP04394H 26569639

[83] O. Link , E. Lugovoy , K. Siefermann , Y. Liu , M. Faubel , and B. Abel , “ Ultrafast electronic spectroscopy for chemical analysis near liquid water interfaces: Concepts and applications,” Appl. Phys. A 96, 117–135 (2009). 10.1007/s00339-009-5179-1

[84] T. Gladytz , B. Abel , and K. R. Siefermann , “ Expansion dynamics of supercritical water probed by picosecond time-resolved photoelectron spectroscopy,” Phys. Chem. Chem. Phys. 17, 4926–4936 (2015). 10.1039/C4CP05171H 25559696

[85] A. Kothe , J. Metje , M. Wilke , A. Moguilevski , N. Engel , R. Al-Obaidi , C. Richter , R. Golnak , I. Y. Kiyan , and E. F. Aziz , “ Time-of-flight electron spectrometer for a broad range of kinetic energies,” Rev. Sci. Instrum. 84, 023106 (2013). 10.1063/1.4791792 23464194

[86] C. A. Arrell , J. Ojeda , M. Sabbar , W. A. Okell , T. Witting , T. Siegel , Z. Diveki , S. Hutchinson , L. Gallmann , U. Keller , F. v. Mourik , R. T. Chapman , C. Cacho , N. Rodrigues , I. C. E. Turcu , J. W. G. Tisch , E. Springate , J. P. Marangos , and M. Chergui , “ A simple electron time-of-flight spectrometer for ultrafast vacuum ultraviolet photoelectron spectroscopy of liquid solutions,” Rev. Sci. Instrum. 85, 103117 (2014). 10.1063/1.4899062 25362381

[87] J. Ojeda , C. A. Arrell , J. Grilj , F. Frassetto , L. Mewes , H. Zhang , F. van Mourik , L. Poletto , and M. Chergui , “ Harmonium: A pulse preserving source of monochromatic extreme ultraviolet (30–110 eV) radiation for ultrafast photoelectron spectroscopy of liquids,” Struct. Dyn. 3, 023602 (2016). 10.1063/1.4933008 26798833 PMC4711517

[88] A. Moguilevski , M. Wilke , G. Grell , S. I. Bokarev , S. G. Aziz , N. Engel , A. A. Raheem , O. Kühn , I. Y. Kiyan , and E. F. Aziz , “ Ultrafast spin crossover in [FeII(bpy)3]2+: Revealing two competing mechanisms by extreme ultraviolet photoemission spectroscopy,” Chem. Phys. Chem. 18, 465–469 (2017). 10.1002/cphc.201601396 28004874

[89] M. Borgwardt , M. Wilke , I. Y. Kiyana , and E. F. Aziz , “ Ultrafast excited states dynamics of [Ru(bpy)3]2+ dissolved in ionic liquids,” Phys. Chem. Chem. Phys. 18, 28893–28900 (2016). 10.1039/C6CP05655E 27722552

[90] N. Engel , S. I. Bokarev , A. Moguilevski , A. A. Raheem , R. Al-Obaidi , T. Möhle , G. Grell , K. R. Siefermann , B. Abel , S. G. Aziz , O. Kühn , M. Borgwardt , I. Y. Kiyan , and E. F. Aziz , “ Light-induced relaxation dynamics of the ferricyanide ion revisited by ultrafast XUV photoelectron spectroscopy,” Phys. Chem. Chem. Phys. 19, 14248–14255 (2017). 10.1039/C7CP01288H 28534587

[91] J. Ojeda , C. A. Arrell , L. Longetti , M. Chergui , and J. Helbing , “ Charge-transfer and impulsive electronic-to-vibrational energy conversion in ferricyanide: Ultrafast photoelectron and transient infrared studies,” Phys. Chem. Chem. Phys. 19, 17052–17062 (2017). 10.1039/C7CP03337K 28650009

[92] M. Borgwardt , M. Wilke , T. Kampen , S. Mähl , M. Xiao , L. Spiccia , K. M. Lange , I. Y. Kiyan , and E. F. Aziz , “ Charge transfer dynamics at dye-sensitized ZnO and TiO_2_ interfaces studied by ultrafast XUV photoelectron spectroscopy,” Sci. Rep. 6, 24422 (2016). 10.1038/srep24422 27073060 PMC4829909

[93] I. Jordan , M. Huppert , M. A. Brown , J. A. van Bokhoven , and H. J. Wörner , “ Photoelectron spectrometer for attosecond spectroscopy of liquids and gases,” Rev. Sci. Instrum. 86, 123905 (2015). 10.1063/1.4938175 26724045

[94] J. M. Schins , P. Breger , P. Agostini , R. C. Constantinescu , H. G. Muller , G. Grillon , A. Antonetti , and A. Mysyrowicz , “ Laser-assisted Auger decay as free-free transitions in a high-intensity laser field,” Phys. Rev. A 52, 1272 (1995). 10.1103/PhysRevA.52.1272 9912366

[95] T. E. Glover , R. W. Schoenlein , A. H. Chin , and C. V. Shank , “ Observation of laser assisted photoelectric effect and femtosecond high order harmonic radiation,” Phys. Rev. Lett. 76, 2468 (1996). 10.1103/PhysRevLett.76.2468 10060707

[96] L. Miaja-Avila , C. Lei , M. Aeschlimann , J. L. Gland , M. M. Murnane , H. C. Kapteyn , and G. Saathoff , “ Laser-assisted photoelectric effect from surfaces,” Phys. Rev. Lett. 97, 113604 (2006). 10.1103/PhysRevLett.97.113604 17025885

[97] Y. H. Wang , H. Steinberg , P. Jarillo-Herrero , and N. Gedik , “ Observation of Floquet-Bloch states on the surface of a topological insulator,” Science 342, 453–457 (2013). 10.1126/science.1239834 24159040

[98] C. A. Arrell , J. Ojeda , L. Mewes , J. Grilj , F. Frassetto , L. Poletto , F. van Mourik , and M. Chergui , “ Laser-assisted photoelectric effect from liquids,” Phys. Rev. Lett. 117, 143001 (2016). 10.1103/PhysRevLett.117.143001 27740777

[99] S. Hüfner , *Photoelectron Spectroscopy: Principles and Applications* ( Springer, 2003).

[100] P. J. Feibelman and D. E. Eastman , “ Photoemission spectroscopy—Correspondence between quantum theory and experimental phenomenology,” Phys. Rev. B 10, 4932 (1974). 10.1103/PhysRevB.10.4932

[101] J. Bokor , “ Ultrafast dynamics at semiconductor and metal surfaces,” Science 246, 1130 (1989). 10.1126/science.246.4934.1130 17820958

[102] O. Björneholm , A. Nilsson , A. Sandell , B. Hernnäs , and N. Mårtensson , “ Determination of time scales for charge transfer screening in physisorbed molecules,” Phys. Rev. Lett. 68, 1892 (1992). 10.1103/PhysRevLett.68.1892 10045247

[103] P. A. Brühwiler , O. Karis , and N. Mårtensson , “ Charge-transfer dynamics studied using resonant core spectroscopies,” Rev. Mod. Phys. 74, 703 (2002). 10.1103/RevModPhys.74.703

[104] W. Wurth and D. Menzel , “ Ultrafast electron dynamics at surfaces probed by resonant Auger spectroscopy,” Chem. Phys. 251, 141 (2000). 10.1016/S0301-0104(99)00305-5

[105] M. Bauer and M. Aeschlimann , “ Dynamics of excited electrons in metals, thin films and nanostructures,” J. Electron. Spectrosc. Relat. Phenom. 124, 225 (2002). 10.1016/S0368-2048(02)00056-7

[106] X. Y. Zhu , “ Electronic structure and electron dynamics at molecule-metal interfaces: Implications for molecule-based electronics,” Surf. Sci. Rep. 56, 1 (2004). 10.1016/j.surfrep.2004.09.002

[107] T. Hertel , E. Knoesel , M. Wolf , and G. Ertl , “ Ultrafast electron dynamics at Cu(111): Response of an electron gas to optical excitation,” Phys. Rev. Lett. 76, 535 (1996). 10.1103/PhysRevLett.76.535 10061481

[108] M. Wolf , A. Hotzel , E. Knoesel , and D. Velic , “ Direct and indirect excitation mechanisms in two-photon photoemission spectroscopy of Cu(111) and CO/Cu(111),” Phys. Rev. B 59, 5926 (1999). 10.1103/PhysRevB.59.5926

[109] N. Pontius , V. Sametoglu , and H. Petek , “ Simulation of two-photon photoemission from the bulk sp-bands of Ag(111),” Phys. Rev. B 72, 115105 (2005). 10.1103/PhysRevB.72.115105

[110] U. Höfer , I. L. Shumay , C. Reuss , U. Thomann , W. Wallauer , and T. Fauster , “ Time-resolved coherent photoelectron spectroscopy of quantized electronic states on metal surfaces,” Science 277, 1480 (1997). 10.1126/science.277.5331.1480

[111] J. Güdde , M. Rohleder , T. Meier , S. W. Koch , and U. Höfer , “ Time-resolved investigation of coherently controlled electric currents at a metal surface,” Science 318, 1287 (2007). 10.1126/science.1146764 18033880

[112] W. L. Yang , J. D. Fabbri , T. M. Willey , J. R. I. Lee , J. E. Dahl , R. M. K. Carlson , P. R. Schreiner , A. A. Fokin , B. A. Tkachenko , N. A. Fokina , W. Meevasana , N. Mannella , K. Tanaka , X. J. Zhou , T. V. Buuren , M. A. Kelly , Z. Hussain , N. A. Melosh , and Z.-X. Shen , “ Monochromatic electron photoemission from diamondoid monolayers,” Science 316, 1460–1462 (2007). 10.1126/science.1141811 17556579

[113] F. J. Himpsel , J. A. Knapp , J. A. Van Vechten , and D. E. Eastman , “ Quantum photoyield of diamond(111)—A stable negative-affinity emitter,” Phys. Rev. B 20, 624 (1979). 10.1103/PhysRevB.20.624

[114] S. Roth , D. Leuenberger , J. Osterwalder , J. E. Dahl , R. M. K. Carlson , B. A. Tkachenko , A. A. Fokin , P. R. Schreiner , and M. Hengsberger , “ Negative-electron-affinity diamondoid monolayers as high-brilliance source for ultrashort electron pulses,” Chem. Phys. Lett. 495, 102–108 (2010). 10.1016/j.cplett.2010.06.063

[115] R. Locher , L. Castiglioni , M. Lucchini , M. Greif , L. Gallmann , J. Osterwalder , M. Hengsberger , and U. Keller , “ Energy-dependent photoemission delays from noble metal surfaces by attosecond interferometry,” Optica 2, 405–410 (2015). 10.1364/OPTICA.2.000405

[116] M. Rohleder , W. Berthold , J. Gu¨dde , and U. Höfer , “ Time-resolved two-photon photoemission of buried interface states in Ar/Cu(100),” Phys. Rev. Lett. 94, 017401 (2005). 10.1103/PhysRevLett.94.017401 15698130

[117] A. L. Cavalieri , N. Müller , T. Uphues , V. S. Yakovlev , A. Baltuska , B. Horvath , B. Schmidt , L. Blümel , R. Holzwarth , S. Hendel , M. Drescher , U. Kleineberg , P. M. Echenique , R. Kienberger , F. Krausz , and U. Heinzmann , “ Attosecond spectroscopy in condensed matter,” Nature 449, 1029–1032 (2007). 10.1038/nature06229 17960239

[118] S. Neppl , R. Ernstorfer , E. M. Bothschafter , A. L. Cavalieri , D. Menzel , J. V. Barth , F. Krausz , R. Kienberger , and P. Feulner , “ Attosecond time-resolved photoemission from core and valence states in magnesium,” Phys. Rev. Lett. 109, 087401 (2012). 10.1103/PhysRevLett.109.087401 23002773

[119] S. Neppl , R. Ernstorfer , A. L. Cavalieri , C. Lemell , G. Wachter , E. Magerl , E. M. Bothschafter , M. Jobst , M. Hofstetter , U. Kleineberg , J. V. Barth , D. Menzel , J. Burgdörfer , P. Feulner , F. Krausz , and R. Kienberger , “ Direct observation of electron propagation and dielectric screening on the atomic length scale,” Nature 517, 342–346 (2015). 10.1038/nature14094 25592539

[120] W. A. Okell , T. Witting , D. Fabris , C. A. Arrell , J. Hengster , S. Ibrahimkutty , A. Seiler , M. Barthelmess , S. Stankov , D. Y. Lei , Y. Sonnefraud , M. Rahmani , T. Uphues , S. A. Maier , J. P. Marangos , and J. W. G. Tisch , “ Temporal broadening of attosecond photoelectron wavepackets from solid surfaces,” Optica 2, 383–387 (2015). 10.1364/OPTICA.2.000383

[121] C.-H. Zhang and U. Thumm , “ Attosecond photoelectron spectroscopy of metal surfaces,” Phys. Rev. Lett. 102, 123601–123601 (2009). 10.1103/PhysRevLett.102.123601 19392274

[122] A. K. Kazansky and P. M. Echenique , “ One-electron model for the electronic response of metal surfaces to subfemtosecond photoexcitation,” Phys. Rev. Lett. 102, 177401 (2009). 10.1103/PhysRevLett.102.177401 19518828

[123] J. C. Baggesen and L. B. Madsen , “ Theory for time-resolved measurements of laser-induced electron emission from metal surfaces,” Phys. Rev. A 78, 032903 (2008). 10.1103/PhysRevA.78.032903

[124] A. G. Borisov , D. Sanchez-Portal , A. K. Kazansky , and P. M. Echenique , “ Resonant and nonresonant processes in attosecond streaking from metals,” Phys. Rev. B 87, 121110 (2013). 10.1103/PhysRevB.87.121110

[125] C. Lemell , B. Solleder , K. Tõkési , and J. Burgdörfer , “ Simulation of attosecond streaking of electrons emitted from a tungsten surface,” Phys. Rev. A 79, 062901 (2009). 10.1103/PhysRevA.79.062901

[126] C.-H. Zhang and U. Thumm , “ Streaking and Wigner time delays in photoemission from atoms and surfaces,” Phys. Rev. A 84, 033401 (2011). 10.1103/PhysRevA.84.033401

[127] U. Heinzmann and J. H. Dil , “ Spin–orbit-induced photoelectron spin polarization in angle-resolved photoemission from both atomic and condensed matter targets,” J. Phys.: Condens. Matter 24, 173001 (2012). 10.1088/0953-8984/24/17/173001 22480989

[128] M. Fanciulli , H. Volfová , S. Muff , J. Braun , H. Ebert , J. Minár , U. Heinzmann , and J. H. Dil , “ Spin polarization and attosecond time delay in photoemission from spin degenerate states of solids,” Phys. Rev. Lett. 118, 067402 (2017). 10.1103/PhysRevLett.118.067402 28234536

[129] Z. Tao , C. Chen , T. Szilvasi , M. Keller , M. Mavrikakis , H. Kapteyn , and M. Murnane , “ Direct time-domain observation of attosecond final-state lifetimes in photoemission from solids,” Science 353, 62 (2016). 10.1126/science.aaf6793 27256880 PMC7586730

[130] M. Lucchini , L. Castiglioni , L. Kasmi , P. Kliuiev , A. Ludwig , M. Greif , J. Osterwalder , M. Hengsberger , L. Gallmann , and U. Keller , “ Light-matter interaction at surfaces in the spatiotemporal limit of macroscopic models,” Phys. Rev. Lett. 115, 137401 (2015). 10.1103/PhysRevLett.115.137401 26451581

